# Peptide barcoding for one-pot evaluation of sequence–function relationships of nanobodies

**DOI:** 10.1038/s41598-021-01019-6

**Published:** 2021-11-02

**Authors:** Yusei Matsuzaki, Wataru Aoki, Takumi Miyazaki, Shunsuke Aburaya, Yuta Ohtani, Kaho Kajiwara, Naoki Koike, Hiroyoshi Minakuchi, Natsuko Miura, Tetsuya Kadonosono, Mitsuyoshi Ueda

**Affiliations:** 1 Division of Applied Life Sciences, Graduate School of Agriculture, Kyoto University, Sakyo-ku, Kyoto, 606-8502 Japan; 2 Kyoto Integrated Science and Technology Bio-Analysis Center, Simogyo-ku, Kyoto, 600-8813 Japan; 3 JST, CREST, Chiyoda-ku, Tokyo, 102-0076 Japan; 4 JST, COI-NEXT, Chiyoda-ku, Tokyo, 102-0076 Japan; 5 JST, FOREST, Chiyoda-ku, Tokyo, 102-0076 Japan; 6 TechnoPro, Inc. TechnoPro R&D, Company, Tokyo, 106-6135 Japan; 7 Kyoto Monotech, Kamigyo-ku, Kyoto, 602-8155 Japan; 8 Graduate School of Life and Environmental Sciences, Osaka Prefecture University, Naka-ku, Sakai, 599-8531 Japan; 9 School of Life Science and Technology, Tokyo Institute of Technology, Midori-ku, Yokohama, 226-8501 Japan

**Keywords:** High-throughput screening, Mass spectrometry, Antibody therapy

## Abstract

Optimisation of protein binders relies on laborious screening processes. Investigation of sequence–function relationships of protein binders is particularly slow, since mutants are purified and evaluated individually. Here we developed peptide barcoding, a high-throughput approach for accurate investigation of sequence–function relationships of hundreds of protein binders at once. Our approach is based on combining the generation of a mutagenised nanobody library fused with unique peptide barcodes, the formation of nanobody–antigen complexes at different ratios, their fine fractionation by size-exclusion chromatography and quantification of peptide barcodes by targeted proteomics. Applying peptide barcoding to an anti-GFP nanobody as a model, we successfully identified residues important for the binding affinity of anti-GFP nanobody at once. Peptide barcoding discriminated subtle changes in *K*_D_ at the order of nM to sub-nM. Therefore, peptide barcoding is a powerful tool for engineering protein binders, enabling reliable one-pot evaluation of sequence–function relationships.

## Introduction

Protein binders are indispensable molecules for therapeutics^1^, diagnostics^2^ and basic science^3^. However, current screening processes of protein binders are labour-intensive. Especially, investigating sequence–function relationships of a protein binder, which is essential for optimising protein binders, is slow because individual mutated binders are separately evaluated using low-throughput technologies^4–6^. Typically, diverse amino-acid-substituted mutants are constructed and purified separately, and their binding kinetics are individually evaluated by enzyme-linked immunosorbent assay^7^, surface plasmon resonance (SPR)^8,9^ or biolayer interferometry^10,11^.

Display technologies, such as phage^12^, yeast^13^, ribosome^14^ and messenger RNA^15^ display, are used as alternatives to low-throughput technologies. These technologies use protein binder display on a surface, which ensures physical genotype–phenotype linkage to retrieve genetic information after selection. Although display technologies enable high-throughput evaluation of highly diverse mutants of a protein binder, fusion of a protein binder on a surface inevitably causes negative effects on screening processes. Display on a surface increases stability of protein binders, and screened protein binders may lose their activities upon conversion into soluble forms^16,17^. Oligomeric antigens can cause avidity, leading to difficulties in accurate estimation of binding kinetics^18,19^. Fusion partners can cause nonspecific binding. For example, phage can nonspecifically bind to antigens^20^ and the huge size of it could lead to co-enrichment of aggregated molecules^21^. Consequently, display technologies are not appropriate for accurate evaluation of sequence–function relationships of protein binders.

Mass spectrometry (MS)-based approaches have been developed to identify protein binders without display on a surface^22–28^. These approaches require proteolytic digestion of protein binder pools and MS analysis of the peptides to estimate binding kinetics. They enable direct detection of free protein binders; however, their potential is limited because (1) unambiguous identification of unique antibody clones is difficult due to the high sequence homology within protein binder pools^27^ and (2) peptides derived from protein binders often exhibit low ionisation efficiencies, resulting in poor detectability^29^.

Recently, Egloff et al. and we developed a peptide barcoding approach for high-throughput, reliable identification of protein binders without being displayed on a surface^8,30^. In this approach, each protein binder is fused with a unique, genetically encoded peptide barcode that contains genotype information. These peptide barcodes are designed to have high detectability for MS, leading to low identification bias and a high identification rate. We used predetermined highly specific, sensitive and quantitative peptide barcodes and detected them using selected reaction monitoring (SRM)-based quantitative targeted proteomics. Egloff et al. used randomised peptide barcodes expected to have high ionisation and fragmentation efficiencies and detected them using an Orbitrap mass spectrometer; they succeeded in deep-mining a nanobody (Nb)-immune repertoire to identify functional protein binders and also in ranking mutant Nbs by their off-rates. These results show the feasibility of peptide barcoding; however, it is unclear whether this approach can accurately evaluate subtle differences in binding kinetics, which is necessary for investigating sequence–function relationships of protein binders.

**Figure 1:**
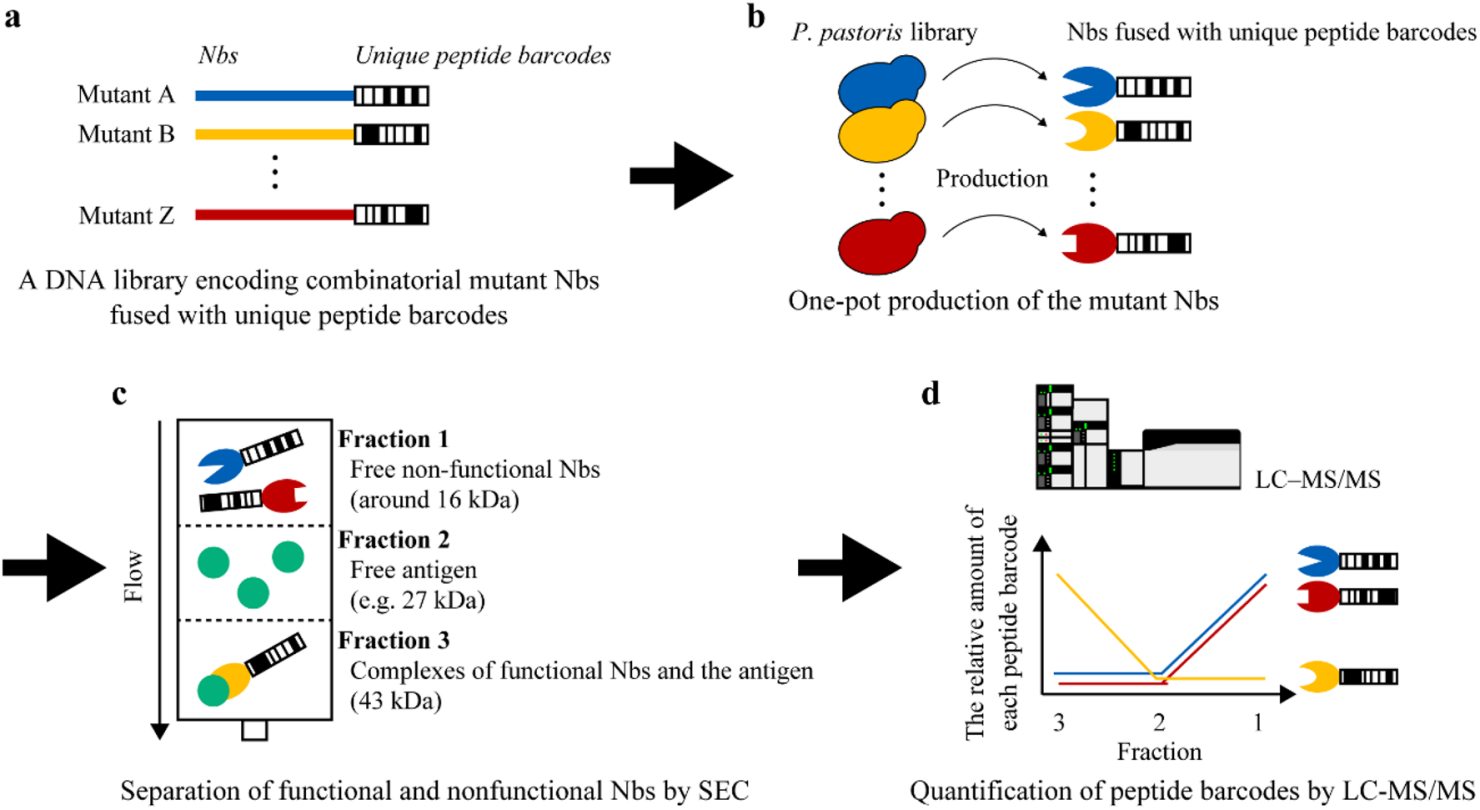
Peptide barcoding for one-pot evaluation of sequence–function relationships of free nanobodies (Nbs). (**a**) DNA library encoding combinatorial mutant Nbs. Each mutant Nb is fused with a unique peptide barcode. (**b**) One-pot production of mutant Nbs by *Pichia pastoris*. (**c**) One-pot separation of functional and nonfunctional Nbs by size-exclusion chromatography (SEC). Each fraction is collected for further analysis. (**d**) Evaluation of sequence–function relationships of free Nbs. Peptide barcodes are cleaved out from Nbs in each fraction and quantified by liquid chromatography–tandem mass spectrometry (LC–MS/MS). The relative amount of each peptide barcode in each fraction correlates to the binding kinetics of each mutant Nb. This figure was created using Illustrator CS2 (https://www.adobe.com/).

Here, we developed peptide barcoding 2.0 for accurate investigation of the binding kinetics of hundreds of free (non-immobilised) Nbs at once (Fig. 1). Our approach is based on combining the generation of a mutagenised Nb library fused with unique peptide barcodes, the formation of Nb–antigen complexes at different ratios, their fine fractionation by size-exclusion chromatography (SEC) and highly specific quantification of peptide barcodes by SRM-based proteomics. In the experiments described below, we demonstrated that peptide barcoding 2.0 is a powerful tool for engineering protein binders, enabling reliable one-pot evaluation of sequence–function relationships.

## Results

### Selection of peptide barcodes

**Figure 2:**
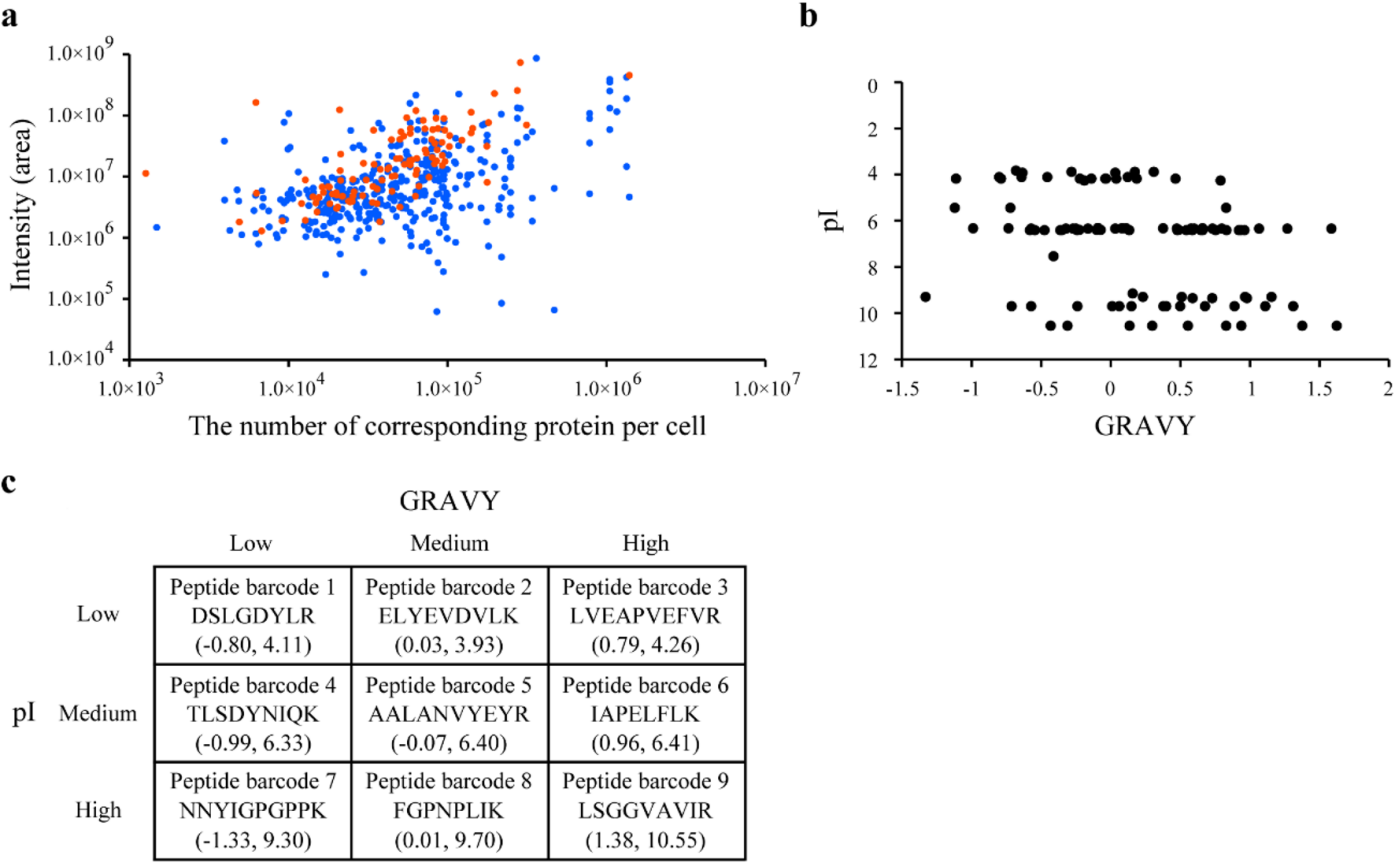
Selection of peptide barcodes. (**a**) Peak intensities of 838 candidate peptide barcodes analysed by liquid chromatography–tandem mass spectrometry (LC–MS/MS). Candidate peptide barcodes were selected using the yeast SRMAtlas. The vertical axis shows the sum of the intensities of each peptide obtained from three independent analyses. The intensity of each peptide was calculated as the sum of the peak areas of four transitions. The horizontal axis shows the number of corresponding proteins per cell calculated previously^35^. Blue dots indicate analysed peptides, and red dots indicate selected peptide barcodes among them. (**b**) Physiochemical properties of the selected 107 peptide barcodes. The hydropathicity (GRAVY) and isoelectric point (pI) of each peptide were mapped in a 2D map. (**c**) Physicochemical properties of nine representative peptide barcodes. The selected 107 peptide barcodes were classified into nine groups based on GRAVY and pI values, and 1 representative peptide was selected from each group. The left and right figures in parentheses indicate GRAVY and pI values, respectively. This figure was created using Illustrator CS2 (https://www.adobe.com/).

We tried to select candidate peptide barcodes from the yeast SRMAtlas^31^, a compendium for highly specific, sensitive and quantitative targeted proteomics. We reasoned that we can easily screen peptides with appropriate profiles as peptide barcodes using the yeast SRMAtlas for SRM-based proteomic workflows. We selected 838 candidate peptides that were composed of 8–10 amino acids without methionine and cysteine^32^ and that showed optimal detectability via liquid chromatography–tandem mass spectrometry (LC–MS/MS). We confirmed that these 838 peptides showed high specificity and detectability in SRM analysis (Fig. 2a and Supplementary Table 1). We selected 107 peptides that are enough to cover all anti-green fluorescent protein (GFP) mutant Nbs with single amino acid substitution. The 107 peptide barcodes had diverse physiochemical properties in terms of the isoelectric point (pI)^33^, hydropathicity (the GRAVY score)^34^ and retention times (Fig. 2b and Supplementary Fig. 1) and showed no sequence similarity (Supplementary Fig. 2). Diverse hydropathicity is especially important to fully exploit the separation power of liquid chromatography (LC).

To determine whether fusion with peptide barcodes affect the function of Nbs, the 107 peptide barcodes were classified into nine categories in terms of hydropathicity and pI, 1 representative peptide was selected from each category (Fig. 2c) and anti-GFP Nb were fused with these representative nine peptide barcodes. We chose anti-GFP Nb as a model because it is a popular Nb used in various studies^36–40^ and the crystal structure of the anti-GFP Nb–GFP complex has been already solved^41,42^, which was an important resource to validate our peptide barcoding analysis. The binding kinetics of these peptide-barcoded Nbs were similar to that of wild-type (WT) Nb (Table 1 and Supplementary Fig. 3). This result indicated that most, if not all, short peptide barcodes with diverse physiochemical properties do not affect the function of anti-GFP Nb.

**Table 1:** Binding kinetics of anti-green fluorescent protein wild-type nanobody fused with peptide barcodes with various physicochemical properties shown in Fig. 2c.

Sample	*k*_a_ (M^−1^ s^−1^)	*k*_d_ (s^−1^)	*K*_D_ (M)
Without peptide barcode	3.64 × 10^6^	1.49 × 10^–4^	4.09 × 10^–11^
With peptide barcode 1	3.75 × 10^6^	1.30 × 10^–4^	3.45 × 10^–11^
With peptide barcode 2	3.16 × 10^6^	1.28 × 10^–4^	4.05 × 10^–11^
With peptide barcode 3	3.23 × 10^6^	1.25 × 10^–4^	3.86 × 10^–11^
With peptide barcode 4	4.10 × 10^6^	1.30 × 10^–4^	3.17 × 10^–11^
With peptide barcode 5	3.06 × 10^6^	1.27 × 10^–4^	4.13 × 10^–11^
With peptide barcode 6	3.52 × 10^6^	1.33 × 10^–4^	3.79 × 10^–11^
With peptide barcode 7	4.41 × 10^6^	1.30 × 10^–4^	2.95 × 10^–11^
With peptide barcode 8	4.38 × 10^6^	1.25 × 10^–4^	2.86 × 10^–11^
With peptide barcode 9	3.63 × 10^6^	1.52 × 10^–4^	4.19 × 10^–11^

### Production of anti-GFP alanine scanning mutant Nbs

We planned to carry out alanine scanning of anti-GFP Nb to prove the feasibility of one-pot evaluation of sequence–function relationships of free Nbs. Alanine scanning is an appropriate method to identify important residues of proteins and provide important information for investigating sequence–function relationships. Each non-alanine residue of anti-GFP Nb was substituted with alanine. We constructed a library of 107 plasmids encoding anti-GFP WT and mutant Nbs fused with unique peptide barcodes at their C-termini (Supplementary Table 2). The library was introduced into *Pichia pastoris* by electroporation. We obtained 2500 unique clones, which were sufficient to cover all 107 anti-GFP WT and mutant Nbs. The colonies were collected in one-pot and subjected to production and purification processes. Successful production and purification of anti-GFP WT and mutant Nbs were confirmed by sodium dodecyl sulphate–polyacrylamide gel electrophoresis (SDS-PAGE) and Coomassie Brilliant Blue (CBB) staining (Supplementary Fig. 4). The purified anti-GFP mutant Nbs were subjected to SRM analysis, and we identified most of anti-GFP mutant Nbs (102/107) (Supplementary Table 3).

### One-pot evaluation of sequence–function relationships of free anti-GFP mutant Nbs using peptide barcoding

**Figure 3:**
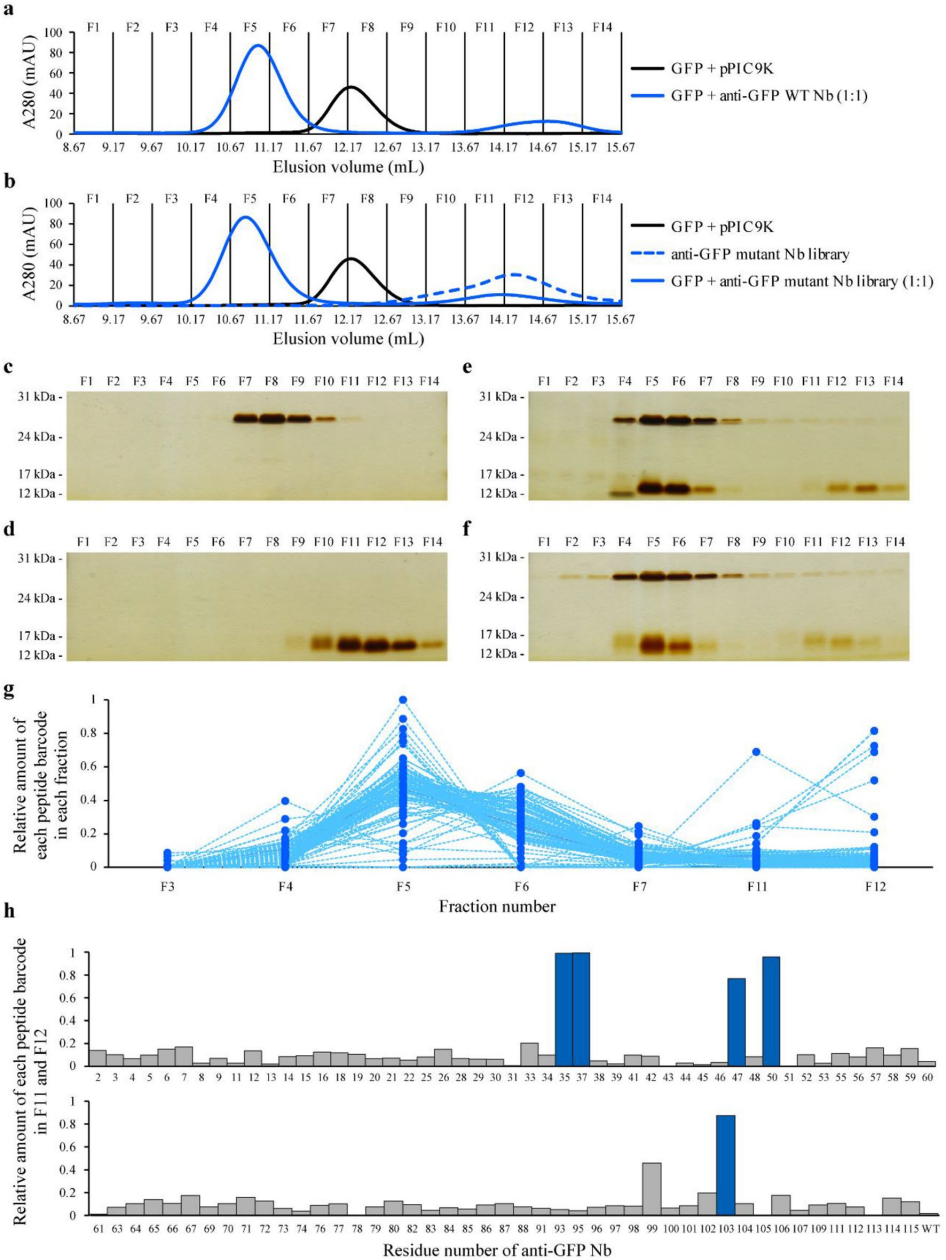
One-pot evaluation of affinities of the anti-green fluorescent protein (GFP) mutant nanobody (Nb) library. (**a**, **b**) Size-exclusion chromatography (SEC) for separation of functional and nonfunctional Nbs. To confirm separation of GFP and the GFP–Nb complex, GFP alone and a mixture of equimolar amounts of GFP and anti-GFP wild-type (WT) Nb were subjected to SEC in (**a**). For one-pot evaluation of affinities of the anti-GFP mutant Nb library, GFP alone, the anti-GFP mutant Nb library alone and a mixture of equimolar amounts of GFP and the anti-GFP mutant Nb library were subjected to SEC in (**b**). The purified sample from *Pichia pastoris* transformed with a backbone vector (pPIC9K) was used as a control. Fourteen fractions were collected in each experiment. (**c**–**f**) Sodium dodecyl sulphate–polyacrylamide gel electrophoresis and silver staining of collected fractions. Fractions from the SEC analysis of GFP (27 kDa) are shown in (**c**), anti-GFP WT Nb (16 kDa) in (d), equimolar amounts of GFP and anti-GFP WT Nb in (**e**) and equimolar amounts of GFP and the anti-GFP mutant Nb library in (**f**). Fraction numbers correspond to those of SEC analysis. These gels are cropped and full-length gels are presented in Supplementary Figs. 9–12. (**g**) Quantification of the relative amount of each peptide barcode in each fraction. The total amount of each peptide barcode in fractions F3–F7 and F11–F12 was defined as 1. Each dotted line indicates each peptide barcode. (**h**) Identification of nonfunctional anti-GFP mutant Nbs. The graph shows the relative amount of each peptide barcode in fractions F11 and F12 in which nonfunctional mutant Nbs were enriched. The total amount of each peptide barcode in fractions F3–F7 and F11–F12 was defined as 1. Five nonfunctional anti-GFP mutant Nbs whose peptide barcodes were mostly detected in fractions F11 and F12 (> 50%) are coloured in dark blue. Anti-GFP mutant Nbs whose peptide barcodes were not identified by mass spectrometry are not shown. The data shown are the first of two independent experiments, and the second showed equivalent results to the first (Supplementary Fig. 5). This figure was created using Illustrator CS2 (https://www.adobe.com/).

We adopted SEC for one-pot separation of functional and nonfunctional anti-GFP mutant Nbs. First, we investigated the separation power of a Superdex 75 increase 10/300 GL column. We injected GFP (27 kDa) with or without equimolar anti-GFP WT Nb (16 kDa) into the column. The GFP peak clearly shifted with the addition of anti-GFP WT Nb to higher-molecular-weight fractions (Fig. 3a and Supplementary Fig. 5a), indicating successful separation of GFP and the anti-GFP WT Nb–GFP complex using SEC. Then, we carried out one-pot separation of functional and nonfunctional anti-GFP mutant Nbs by SEC. GFP alone, the anti-GFP mutant Nb library alone and an equimolar mixture of them were separately injected into the column (Fig. 3b and Supplementary Fig. 5b). Injection of anti-GFP mutant Nbs alone showed a single peak, suggesting that they were purified without aggregation. As shown in Fig. 3a, the GFP peak clearly shifted with the addition of the anti-GFP mutant Nb library to higher-molecular-weight fractions (Fig. 3b). The peak of the complex formed by Nb mutants was slightly shifted from the peak formed by WT Nb probably because of experimental errors derived from AKTA. The fractions (F1–F14) in these SEC experiments were collected and analysed by SDS-PAGE and silver staining (Fig. 3c–f). SDS-PAGE analysis showed that anti-GFP mutant Nbs were separated in two regions, indicating that fractions F4–F7 contained Nbs bound to GFP and fractions F11–F13 contained nonbound Nbs.

Functional and nonfunctional anti-GFP mutant Nbs were annotated by quantification of peptide barcodes derived from Nbs in the collected fractions. The proteins in each fraction were digested and the resultant peptides subjected to SRM analysis. The relative amount of each peptide barcode in each fraction was calculated against the total amount of each peptide barcode in all analysed fractions. The plot of the relative amount of each peptide barcode in each fraction showed that the majority of peptide barcodes were enriched in fraction F5 (Fig. 3g and Supplementary Fig. 5c). This result was consistent with SEC and SDS-PAGE analyses (Fig. 3b,f). No peaks of peptide barcodes were detected in the fractions of the control sample (GFP + pPIC9K in Fig. 3b), suggesting that SRM analysis enables highly specific quantification of peptide barcodes derived from anti-GFP mutant Nbs. To identify nonfunctional mutants, we explored the relative amount of each peptide barcode in the fractions containing nonbound Nbs (F11–F12). Peptide barcodes derived from five anti-GFP mutant Nbs (R35A, Y37A, W47A, G50A and E103A) were highly enriched in the nonbound fractions (Fig. 3h and Supplementary Fig. 5d). More than 90% of the peptide barcodes derived from R35A, Y37A, G50A and E103A and nearly 80% from W47A were found in the nonbound fractions (Fig. 3h). These results suggested that the five anti-GFP mutant Nbs have lower affinities against GFP than that of anti-GFP WT Nb and the difference in the relative amount of each peptide barcode in the nonbound fractions reflected differences in their affinities.

### Stringent enrichment of nonfunctional anti-GFP mutant Nbs

**Figure 4:**
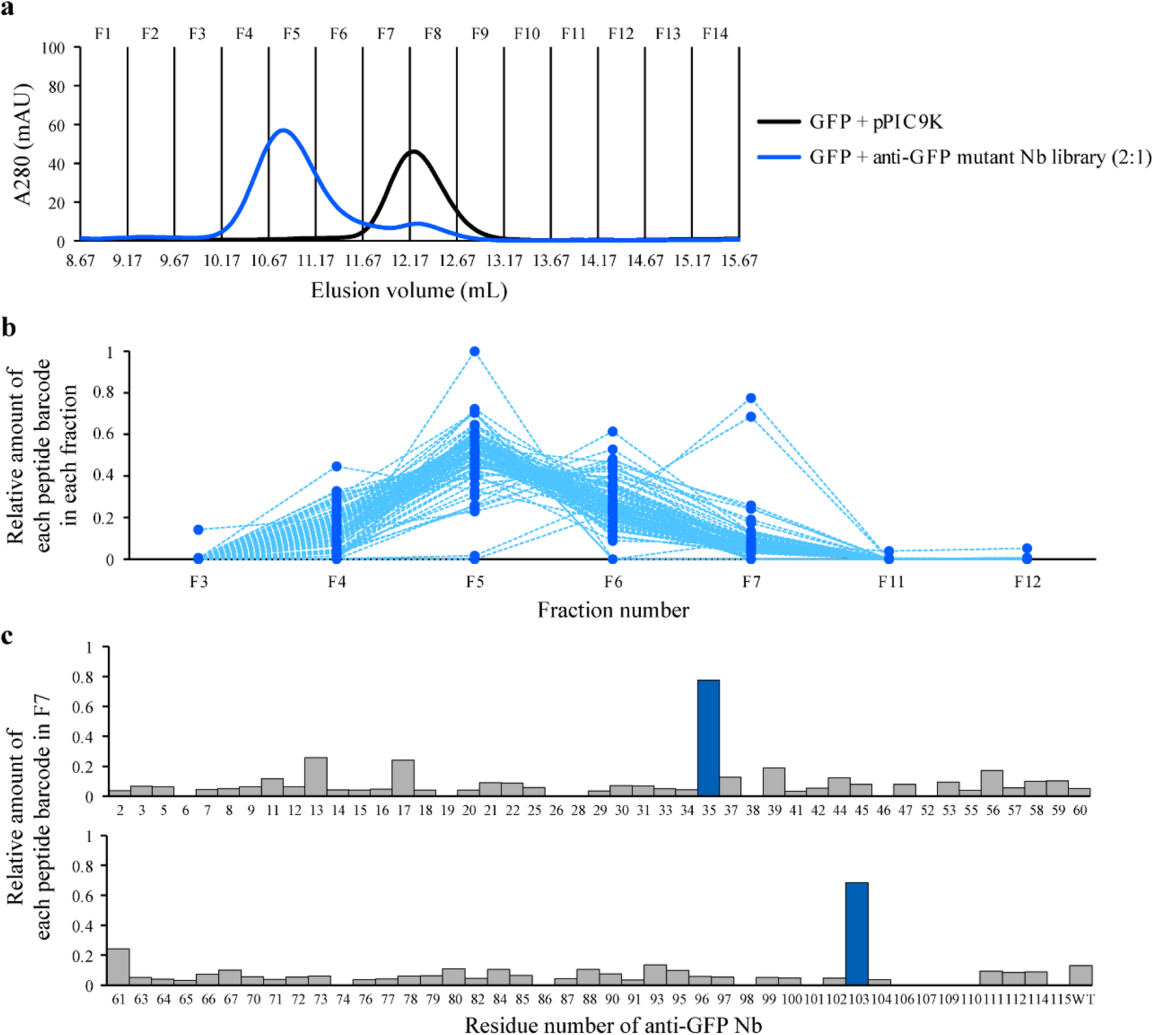
Separation of low-affinity and nonfunctional nanobodies (Nbs) by varying the green fluorescent protein (GFP)/Nb molar ratio. (**a**) Size-exclusion chromatography (SEC) for separation of low-affinity and nonfunctional Nbs. For one-pot evaluation of affinities of the anti-GFP mutant Nb library, GFP alone and a mixture of GFP and the anti-GFP mutant Nb library (2:1 molar amount) were subjected to SEC. The purified sample from *Pichia pastoris* transformed with a backbone vector (pPIC9K) was used as a control. Fourteen fractions were collected in each experiment. (**b**) Quantification of the relative amount of each peptide barcode in each fraction. The total amount of each peptide barcode in fractions F3–F7 was defined as 1. Each dotted line indicates each peptide barcode. (**c**) Identification of nonfunctional anti-GFP mutant Nbs. The graph shows the relative amount of each peptide barcode in fraction F7 in which nonfunctional mutant Nbs were enriched. The total amount of each peptide barcode in fractions F3–F7 was defined as 1. Two nonfunctional anti-GFP mutant Nbs whose peptide barcodes were mostly detected in fraction F7 (> 50%) are coloured in dark blue. Anti-GFP mutant Nbs whose peptide barcodes were not identified by mass spectrometry (including G50A) are not shown. The data shown are the first of two independent experiments, and the second showed equivalent results to the first (Supplementary Fig. 6). This figure was created using Illustrator CS2 (https://www.adobe.com/).

We reasoned that we could separate low-affinity and nonfunctional Nbs by increasing the amount of GFP. We changed the stoichiometric balance of GFP and the anti-GFP mutant Nb library from 1:1 to 2:1 and subjected the protein mixture to SEC. Most of the anti-GFP mutant Nbs were detected in the first half of the fractions (Fig. 4a and Supplementary Fig. 6a), suggesting that most anti-GFP mutant Nbs are bound to GFP. The SEC analysis fractions were collected, and the peptide barcodes in each fraction were quantified by SRM analysis. The plot of the relative amount of each peptide barcode in each fraction showed that the majority of peptide barcodes had the strongest intensities in fraction F5 (Fig. 4b and Supplementary Fig. 6b). Almost no peptide barcode was detected in fractions F11 and F12, suggesting that most Nbs bound to GFP and were eluted at higher-molecular-weight fractions. To identify low-affinity or nonfunctional mutants, we determined the relative amount of each peptide barcode in fraction F7. Peptide barcodes derived from two anti-GFP mutant Nbs (R35A and E103A) were highly enriched in fraction F7 (Fig. 4c and Supplementary Fig. 6c), and 78% of peptide barcodes derived from R35A and 68.5% from E103A were detected in fraction F7 (Fig. 4c). Other peptide barcodes were not enriched in fraction F7. These results suggested that R35A and E103A have very weak or no affinity against GFP.

### Validation of peptide barcoding analyses by SPR analysis

We tried to validate the peptide barcoding analyses by SPR analysis. Five anti-GFP mutant Nbs (R35A, Y37A, W47A, G50A and E103A) without peptide barcodes were produced by *P. pastoris* and purified with Ni–nitrilotriacetic acid (Ni–NTA) agarose. SPR analysis showed that R35A, G50A and E103A did not bind to GFP and Y37A and W47A had lower affinities compared with anti-GFP WT Nb (Table 2 and Supplementary Fig. 7). This result was highly consistent with our peptide barcoding analyses. The anti-GFP mutant Nbs annotated as nonfunctional protein binders in the stringent SEC analysis showed no binding to GFP in SPR. In addition, anti-GFP mutant Nbs annotated as weak protein binders in the first SEC analysis showed weak affinities against GFP in SPR. These results showed that peptide barcoding 2.0 is useful for evaluating the affinities of free Nbs in a multiplex manner.

**Table 2:** Binding kinetics of anti-green fluorescent protein mutant nanobodies identified to have decreased affinities by peptide barcoding.

Sample	*k*_a_ (M^−1^ s^−1^)	*k*_d_ (s^−1^)	*K*_D_ (M)
Wild-type	1.04 × 10^7^	1.29 × 10^–4^	1.24 × 10^–11^
R35A	No binding		
Y37A	4.62 × 10^6^	7.59 × 10^–2^	1.64 × 10^–8^
W47A	8.39 × 10^6^	6.06 × 10^–3^	7.22 × 10^–10^
G50A	No binding		
E103A	No binding		

These mutants were not fused with peptide barcodes.

### Mechanistic estimation of sequence–function relationships of anti-GFP Nb

**Figure 5:**
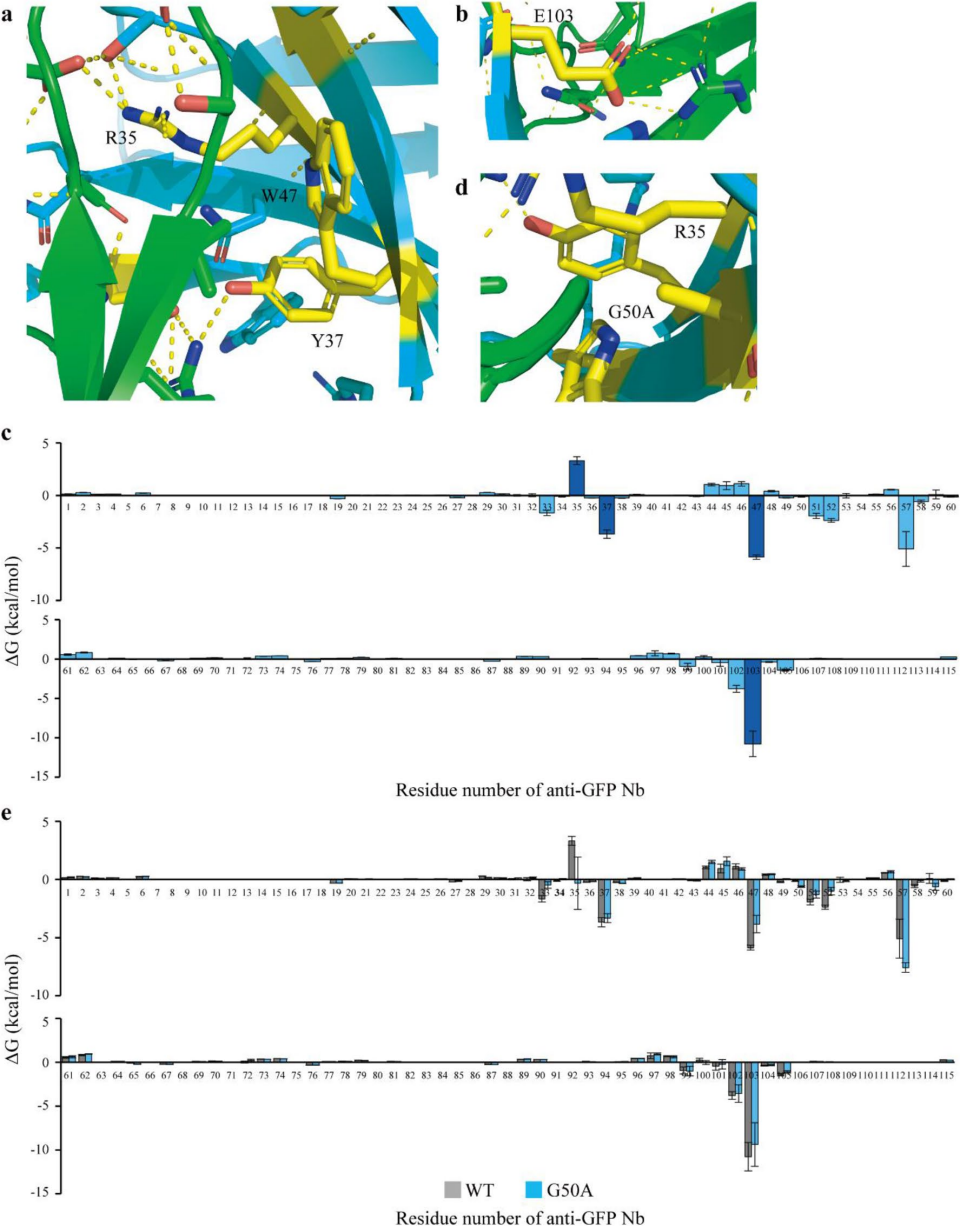
Mechanistic estimation of effects of important residues on binding affinities. (**a**, **b**) Crystal structure of the green fluorescent protein (GFP)–anti-GFP wild-type (WT) Nb complex (PDBID 3OGO)^42^. GFP is coloured in green and Nb in light blue and yellow. The GFP–anti-GFP WT Nb interface was enlarged. (**c**) Calculation of the binding free energy of anti-GFP WT Nb. The binding free energy of each residue against GFP was calculated and shown as the mean ± standard error of the mean (SEM) (*n* = 5) from 50 ns of the prediction run. The important residues annotated by peptide barcoding are shown in dark blue. (**d**) Simulated structure of anti-GFP G50A mutant Nb binding to GFP. The GFP–anti-GFP G50A mutant Nb interface was enlarged. GFP is coloured in green and Nb in light blue and yellow. (**e**) Calculation of the binding free energy of the simulated anti-GFP G50A mutant Nb (light blue). The binding free energy of each residue against GFP was calculated and shown as the mean ± SEM (*n* = 5) from 50 ns of the prediction run. The data of anti-GFP WT Nb are shown in grey for comparison. This figure was created using Illustrator CS2 (https://www.adobe.com/).

We investigated the crystal structure of the anti-GFP Nb–GFP complex (Protein Data Bank accession codes 3OGO [PDBID 3OGO])^42^ to validate our results and to infer why the identified mutations (R35A, Y37A, W47A, G50A and E103A) led to decreased affinities. The crystal structure showed that four residues (R35, Y37, W47 and E103) among the five identified ones are located at the binding surface to GFP^42^ (Fig. 5a,b). We also calculated the binding free energy of the anti-GFP Nb–GFP complex (Fig. 5c). The three residues (Y37, W47 and E103) had high negative ΔG and stabilised interaction with GFP. R35 had positive ΔG, but it forms a salt bridge with E142 of GFP and contributes to specific binding to GFP^42^. The crystal structure and binding free energy analyses supported the results of our peptide barcoding analyses.

Interestingly, G50 was not located at the binding surface to GFP (Fig. 5d), and the binding free energy of G50 was estimated to be almost zero (Fig. 5c). To determine why anti-GFP G50A mutant Nb showed low-affinity, we calculated the binding free energy of a simulated structure of anti-GFP G50A mutant Nb. The binding free energy of R35, an important residue for the specific binding, fluctuated (Fig. 5e). The crystal structure showed that the methyl group of the G50A side chain could change the configuration of R35, leading to decreased affinity (Fig. 5d).

Binding free energy analysis also suggested that R57 and F102 might be important for the function of anti-GFP Nb (Fig. 5c), while peptide barcoding analyses showed no remarkable changes in affinity (Figs. 3h and 4c). We evaluated the affinities of purified anti-GFP R57A and F102A mutant Nbs using SPR and found that they have similar affinities as anti-GFP WT Nb (Table 3 and Supplementary Fig. 8), indicating that peptide barcoding 2.0 can provide highly reliable and multiplex evaluation of the affinities of free Nbs.

**Table 3:** Binding kinetics of anti-green fluorescent protein mutant nanobodies with R57A or F102A mutations.

Sample	*k*_a_ (M^−1^ s^−1^)	*k*_d_ (s^−1^)	*K*_D_ (M)
Wild-type	1.04 × 10^7^	1.29 × 10^–4^	1.24 × 10^–11^
R57A	3.42 × 10^6^	6.72 × 10^–5^	2.00 × 10^–11^
F102A	5.20 × 10^6^	6.28 × 10^–4^	1.21 × 10^–10^

These mutants were not fused with peptide barcodes.

## Discussion

In this study, we successfully developed peptide barcoding 2.0, a simple, fast, quantitative, and reliable approach for investigating sequence–function relationships of hundreds of free Nbs at once. Using anti-GFP Nb as a model, we identified five important residues for target binding (R35, Y37, Y47, G50 and E103). The result was validated by SPR analysis and the crystal structure of the anti-GFP Nb–GFP complex (PDBID 3OGO)^42^. The identified residues R35, Y37, Y47 and E103 are located at the anti-GFP Nb–GFP interface. Binding free energy analysis showed that the residues Y37, W47 and E103 have high negative ΔG for complex formation. Binding free energy analysis also suggested that R57 and F102 might be important for target binding, while peptide barcoding and SPR analyses of these two mutants showed no remarkable changes in affinity. This result indicated that peptide barcoding 2.0 can provide highly reliable and multiplex evaluation of the affinities of free Nbs.

Peptide barcoding 2.0 successfully discriminated subtle change in *K*_D_ at the order of nM to sub-nM. NestLink^30^ enables ranking of mutant Nbs by their off-rates using an antigen trap column. However, the difference in off-rates is evaluated in a narrow range of 10^−2^–10^−3^ s^−1^. Nbs used for research tools or diagnostics have *K*_D_ of the order of nM to sub-nM, and their off-rates are often less than 10^−3^ s^−1^^2,43,44^. This motivated us to develop an improved peptide barcoding methodology to discriminate subtle change in *K*_D_ of the order of nM to sub-nM. Peptide barcoding 2.0 identified five mutants, and follow-up SPR analysis showed that our methodology successfully distinguished anti-GFP WT Nb (*K*_D_ ≈ 10^−11^ M), weakly attenuated mutants (Y37A, *K*_D_ ≈ 10^−8^ M; W47A, *K*_D_ ≈ 10^–10^ M) and nonfunctional mutants (R35A, G50A and E103A). This result indicated that peptide barcoding 2.0 can be useful for Nb engineering.

Peptide barcoding 2.0 has advantages and disadvantages compared to conventional methods. In comparison with display technologies, peptide barcoding 2.0 enables direct evaluation of free protein binders but has limited throughput. Compared to ELISA and SPR, peptide barcoding 2.0 enables a highly multiplex evaluation of mutants in a single analysis. Taking these points into account, peptide barcoding 2.0 is a useful method for accurate evaluation of free binders at a moderate throughput.

We focused on proving peptide barcoding can accurately evaluate subtle differences in binding kinetics, however, it will be essential to improve the scalability of peptide barcoding to enable high-throughput evaluation of a large number of clones for binder selection. Peptide barcoding 2.0 is a potentially scalable method that can evaluate more than 10^5^ clones. More than 100,000 peptides for highly specific, sensitive and quantitative targeted proteomics are ready to use in the SRMAtlas^31,45^. Regarding library construction, DropSynth^46,47^ is used to synthesise thousands of genes at once. Alternatively, when the library size is limited compared with the number of available peptide barcodes, library nesting, in which each Nb gene is linked to numerous unique peptide barcodes in a controlled manner, is used^30^. In library nesting, the linked peptide barcodes are unique because the experimental peptide barcode diversity largely exceeds the number of linked Nbs, enabling unambiguous identification of Nbs. As the library size increases, we need higher separation power of LC. In this case, highly reproducible micro-pillar array columns (µPAC™) may be a better choice^48^.

There are potential biases in peptide barcoding 2.0. For example, differences in production levels of nanobody mutants could lead to identification biases. In this study, we detected 95.3% of peptide barcodes (102/107) in the SEC fractions, indicating some mutants were not produced enough by *P. pastoris*. Concomitant use of other production hosts such as mammalian cells or *E. coli* may mitigate the production bias. In addition, there are potential biases in trypsin digestion and ionization efficiencies. These biases can be mitigated by using tandem Lys-C/trypsin proteolysis^49^ and predetermined peptide barcodes with high specificity and sensitivity^45^.

Peptide barcoding 2.0 and NestLink have some resemblances. Both methods enrich free target protein binders using SEC and detect peptide barcodes by MS spectrometer. These strategies enable a functional evaluation of free binders. However, these methods have some differences. Peptide barcoding 2.0 collects fractions more finely, leading to the detection of subtle differences in affinities among binder mutants. Peptide barcoding uses predetermined peptide barcodes and NestLink randomized peptide barcodes. Predetermined peptide barcodes enable highly selective, sensitive and quantitative analysis of peptide barcodes. Randomized peptide barcodes do not necessarily guarantee selective, sensitive and quantitative analysis but enable more scalable detection of peptide barcodes.

In conclusion, we successfully developed peptide barcoding 2.0, a simple, fast, quantitative, and reliable approach to investigating sequence–function relationships of hundreds of free Nbs at once, which is difficult by conventional low-throughput technologies, where individual protein binders are separately evaluated. Our methodology is also applicable to affinity maturation of Nbs because it can detect subtle differences in affinities of the order of nM to sub-nM, which is often required for diagnostic reagents or drugs. In addition, our methodology is applicable to not only protein binders but also other types of proteins; for example, it will be possible to screen proteins with different profiles, which leads to differences in mobility in SEC analysis.

## Methods

### Selection of candidate peptide barcodes

We selected 12,038 candidate peptides for peptide barcodes from the yeast SRMAtlas^31^. Of these, 838 peptides composed of 8–10 amino acid residues with strong intensities and optimal detectability in the previous LC–MS/MS analysis were selected^31,32^. Peptides containing methionine or cysteine were excluded because of possible oxidation and cross-linking^32^.

### Protein extraction from yeast

We analysed the *Saccharomyces cerevisiae* BY4741 proteome to validate the yeast SRMAtlas data. Briefly, *S. cerevisiae* BY4741 was cultured in yeast extract peptone dextrose (YPD) medium until the optical density at 600 nm (OD_600_) was 1. Three methods were used to lyse yeast cells. First, cells were suspended in lysis buffer A (100 mM NaOH, 50 mM ethylenediaminetetraacetic acid [EDTA], 2% SDS) and incubated at 90 °C for 10 min. Second, cells were suspended in lysis buffer B (100 mM NaCl, 1 mM EDTA, 2% SDS, 10 mM Tris–HCl, pH 8) and disrupted with sonication using a Bioruptor 2 ultrasonic crusher (Sonic Bio Co., Ltd., Kanagawa, Japan). Third, cells were suspended in lysis buffer B and disrupted with grass beads using a shaker. These disrupted cell solutions were centrifuged at 16,000×*g* for 20 min, and proteins were extracted by methanol/chloroform precipitation.

### Protein reduction, alkylation and digestion

Protein reduction, alkylation and digestion were conducted using a phase transfer surfactant^50^. Briefly, the extracted proteins were diluted with solubilise buffer (12 mM sodium deoxycholate, 12 mM *N*-lauroylsarcosinate, 50 mM tetraethylammonium bromide [TEAB]) to obtain 100 μL of 1 mg/mL protein solution. The solution was reduced using dithiothreitol (DTT) (final concentration 50 mM) at 37 °C for 30 min and then alkylated by adding iodoacetamide (final concentration 50 mM). The solution was further diluted five times using 50 mM TEAB and digested with lysyl endopeptidase (FUJIFILM Wako Pure Chemical Corporation, Osaka, Japan) and trypsin (Promega Corporation, Madison, WI, USA) at 37 °C overnight for proteins extracted from yeast and the trypsin/Lys-C Mix, Mass Spec Grade (Promega Corporation) at 37 °C overnight for fractionated Nbs. The detergents were removed with ethyl acetate containing 0.5% trifluoroacetic acid and the resultant solutions were freeze-dried, desalted using MonoSpin C18 (GL Sciences Inc., Tokyo, Japan) and lyophilised. The dried pellets were re-solved with 50 μL of 0.1% formic acid for proteins extracted from yeast and 50 μL of 0.1% formic acid and 0.005% polyethylene glycol 20,000^51^ for fractionated Nbs and then filtered by Ultrafree-MC-HV Centrifugal Filters Durapore PVDF 0.45 μm (Merck Millipore, Burlington, MA, USA). Finally, 1 μL of the aliquot was subjected to LC–MS/MS analysis for proteins extracted from yeast and 5 μL for fractionated Nbs.

### LC–MS/MS analysis

Peptides were analysed by an UltiMate 3000 LC (Thermo Fisher Scientific, Waltham, MA, USA) and LC–MS-8060 triple quadrupole mass spectrometer (Shimadzu Corporation, Kyoto, Japan) system equipped with a C18 monolithic silica capillary column (50 cm, 100 μm internal diameter). Peptides were separated by reverse-phase chromatography using the C18 column, which was kept at 40 °C, with a flow rate of 500 nL/min and injected into the MS system through a nano-electrospray ion source. A gradient was generated by changing the mixing ratio of the two eluents: A (0.1% [v/v] formic acid) and B (acetonitrile containing 0.1% [v/v] formic acid). The gradient programme was as follows: 5% B for 5 min, 5–45% B for 25 min, 45–95% B for 10 min, 95% B for 10 min, and 5% B for 10 min. The heat block and desolvation line temperatures were set at 200 °C and 250 °C, respectively. SRM methods for the peptides were constructed using Skyline (Supplementary Tables S1 and S2)^52^. During peptide barcode selection, four transitions were analysed with a 0.055 m/*z* permissible error. The loop time and dwell time were 8.78–11.34 s and 4–28 ms, respectively. During peptide barcode analyses, four transitions per peptide were analysed, and these transitions were scheduled in a 2 min window before and after the obtained retention time from the analysis of *S. cerevisiae* BY4741 with a 0.055 m/*z* permissible error. The loop time and dwell time were 1.47 s and 5 ms, respectively.

### Purification of anti-GFP Nbs for SPR analysis

DNA fragments encoding anti-GFP WT Nb or anti-GFP mutant Nbs fused with or without peptide barcodes were inserted into the pPIC9K_6 × His vector (Supplementary Table 2 and “Supplementary Information”) digested by *Eco*RI and *Spe*I using the In-Fusion^®^ HD Cloning Kit (Takara Bio Inc., Shiga, Japan). These plasmids were digested by *Sac*I, column-purified and introduced into the *P. pastoris* GS115 strain (Thermo Fisher Scientific) using the Frozen EZ Yeast Transformation II Kit (Zymo Research Corporation, Irvine, CA, USA). Transformants were selected on MD solid medium and cultured in 20 mL of buffered glycerol-complex medium (BMGY) at 30 °C for 48 h, and the cells were inoculated in 10 mL of buffered methanol-complex medium (BMMY) and cultured at 30 °C for 24 h. The culture media were filtered using a 0.45 μm filter. The Nbs obtained were purified using His SpinTrap™ (Cytiva, Marlborough, MA, USA). The solvent of the purified solutions was replaced with HBS-EP + buffer (Cytiva) using Amicon Ultra-0.5 Centrifugal Filters Ultracel-3K (Merck Millipore). The purity of Nbs was confirmed using SDS-PAGE and CBB staining, and their concentrations were estimated with *A*_280_ extinction coefficients calculated using Benchling software (Benchling, San Francisco, CA, USA).

### GFP production in *Escherichia coli*

GFP was produced by *E. coli* BL21 (DE3) harbouring the *GFP* gene. The cells were cultured in 80 mL of LBK medium at 37 °C until OD_600_ was 0.6. Isopropyl-β-d-thiogalactopyranoside was added at a final concentration of 0.1 M, and the cells were further incubated for 4 h. Next, the cells were disrupted by sonication using a Bioruptor 2 (Sonic Bio Co., Ltd.), and the supernatant was incubated with 100 μL of Ni–nitrilotriacetic acid (NTA) agarose (FUJIFILM Wako Pure Chemical Corporation). GFP was eluted with 200 μL of elution buffer containing 250 mM imidazole. The purity of GFP was confirmed using SDS-PAGE and CBB staining, and its concentration was estimated with *A*_280_ extinction coefficients calculated using Benchling software.

### Surface plasmon resonance

Kinetic parameters of Nbs were measured using Biacore T-200 (Cytiva) with the multicycle method. According to the manufacturer’s protocol, 265–274 response units of recombinant GFP were immobilised on Series S Sensor Chip CM5 (Cytiva). Solutions containing anti-GFP Nbs were diluted with HBS-EP + buffer to prepare five diluted series. The flow rate and temperature were 30 μL/min and 25 °C, respectively, and the running buffer was HBS-EP + buffer. Contact time and dissociation time varied depending on the measurements. The GFP-coated chip was regenerated using 50 mM NaOH (flow rate 30 μL/min and contact time 20 s).

### Preparation of the anti-GFP mutant Nb library for SEC analysis

We constructed plasmids encoding peptide-barcoded anti-GFP Nb mutants with single alanine substitutions by primer-based mutagenesis. In brief, two complement primers were designed to introduce alanine (GCT codon) substitution at the target residue. In addition, one primer encoding unique peptide barcode was designed to anneal the 3′ end of anti-GFP Nb and another primer to anneal the 5′ end of anti-GFP Nb. PCR was performed using these four primers to prepare two DNA fragments, and the fragments were inserted into the pPIC9K_6 × His vector digested by *Eco*RI and *Spe*I using the In-Fusion® HD Cloning Kit (Takara Bio Inc.). A plasmid library containing all mutant plasmids was transformed into the *P. pastoris* GS115 strain, as previously described^53^. Briefly, the plasmid library was digested by *Sac*I and purified to a concentration of 5 ng/μL. *P. pastoris* was grown in YPD medium until OD_600_ was 1.5, and 8 × 10^8^ cells were collected, suspended in a buffer (100 mM lithium acetate, 10 mM DTT, 600 mM sorbitol and 10 mM Tris–HCl, pH 7.5) and incubated at room temperature for 30 min. Next, the cells were washed thrice with ice-cold 1 M sorbitol and resuspended (~ 10^10^ cells/mL) in 80 μL of 1 M sorbitol containing 20 ng of purified DNA fragments. The cells were then transferred to a 0.2 cm gap vial, and a pulse was applied at 1.5 kV, 25 µF and 200 Ω using the Gene Pulser^®^ II Electroporation System (Bio-Rad Laboratories, Inc., Hercules, CA, USA). The cells were immediately diluted with 1 mL of ice-cold 1 M sorbitol, inoculated in 10 mL of SC-His medium and cultured at 30 °C overnight. The transformants were selected on RDB solid medium. The transformation efficiency was calculated from RDB solid medium on which 20 μL aliquots were plated and incubated at 30 °C for 3 days. The colonies were collected with 20 mL of BMGY and cultured at 30 °C for 24 h. Next, cells in 1 mL of the culture medium were stored at − 80 °C with an equal amount of 30% glycerol. The glycerol stock was thawed, and the cells were suspended in 100 mL of BMGY for Nb production. After cultivation at 30 °C for 24 h, the cells were transferred to 50 mL of BMMY and cultured at 30 °C for 24 h. The culture supernatant was collected and filtered using a 0.45 μm filter. Nbs in the supernatant were adsorbed on 250 μL of Ni–NTA agarose (FUJIFILM Wako Pure Chemical Corporation) and eluted with 500 μL of elution buffer containing 250 mM imidazole. Finally, the purity of Nbs was confirmed using SDS-PAGE and CBB staining, and their concentration was estimated with *A*_280_ extinction coefficients calculated using Benchling software.

### SEC analysis

SEC analysis was conducted using ÄKTAexplorer 10S (Cytiva) equipped with a Superdex 75 increase 10/300 GL column (Cytiva). A mixture of GFP and anti-GFP Nbs (2 nmol each of GFP and anti-GFP Nbs in Fig. 3 and 2 nmol of GFP and 1 nmol of anti-GFP Nbs in Fig. 4) in phosphate-buffered saline (PBS, pH 7.4) was prepared to a total volume of 400 μL and injected into the column. Next, six-fifth of the column volume of PBS (pH 7.4) was loaded at a flow rate of 0.8 mL/min. The eluate was collected in 0.5 mL portions. Next, 10 μL of the collected fractions were subjected to SDS-PAGE and silver staining using Sil-Best Stain One (Nacalai Tesque, Inc., Kyoto, Japan). Finally, the fractionated solutions were lyophilised and stored at − 80 °C.

### Binding free energy analysis

The initial coordinates of the anti-GFP Nb–GFP complex were obtained from PDBID 3OGO^42^. The G50A structure was generated by introducing a point mutation into the Nb structure. All molecular dynamics simulations were performed using the AMBER 16 program^54^ on TSUBAME (Global Scientific Information and Computing Center, Tokyo Institute of Technology, Japan). For binding free energy calculations using the anti-GFP Nb–GFP complex, the systems were fully solvated with explicit solvent and six Na^+^ counterions were added to obtain electrostatic neutrality. We used the AMBER ff14SB force field for proteins and the TIP3P model for water molecules. Next, the systems were optimised by energy minimisation and equilibrated with backbone restraints. Production runs were performed for 100 ns. The binding free energy of anti-GFP Nbs and GFP during the final 50 ns of the production run was calculated using the molecular mechanics/generalised Born surface area (MM/GBSA) module.

## Supplementary Information

Supplementary Tables.

Supplementary Figures.

## Data Availability

MS data generated and/or analysed in this study are available in the jPOST repository^55^ (JPST001198). Sequences of plasmids used in the study are shown in Supplementary Information. The other datasets generated and analysed during the current study are available from the corresponding author on reasonable request.
