# Supplementary material for: Peptide barcoding for one-pot evaluation of sequence–function relationships of nanobodies
Source: Sci Rep. 2021 Nov 2;11:21516. doi: 10.1038/s41598-021-01019-6 (PMC8563947; doi:10.1038/s41598-021-01019-6)
Supplement: Supplementary file 2 — Supplementary Figures. [file 41598_2021_1019_MOESM2_ESM.pdf]

Peptide barcoding for one-pot evaluation of sequence–function relationships of nanobodies

Yusei Matsuzaki[1], Wataru Aoki[1,2,3,4,5*], Takumi Miyazaki[1], Shunsuke Aburaya[1], Yuta Ohtani[1], Kaho Kajiwara[1], Naoki Koike[6], Hiroyoshi Minakuchi[7], Natsuko Miura[8], Tetsuya Kadonosono[9], Mitsuyoshi Ueda[1,2,3,4]

[1]Division of Applied Life Sciences, Graduate School of Agriculture, Kyoto University, Sakyo-ku, Kyoto, 606-8502, Japan

[2]Kyoto Integrated Science and Technology Bio-Analysis Center, Simogyo-ku, Kyoto, 600-8813, Japan

[3]JST, CREST, Chiyoda-ku, Tokyo, 102-0076, Japan

[4]JST, COI-NEXT, Chiyoda-ku, Tokyo, 102-0076, Japan

[5]JST, FOREST, Chiyoda-ku, Tokyo, 102-0076, Japan

[6]TechnoPro, Inc. TechnoPro R&D, Company, Tokyo, 106-6135, Japan

[7]Kyoto Monotech, Kamigyo-ku, Kyoto, 602-8155, Japan

[8]Graduate School of Life and Environmental Sciences, Osaka Prefecture University, Naka-ku, Sakai, 599-8531, Japan

[9]School of Life Science and Technology, Tokyo Institute of Technology, Midori-ku, Yokohama, 226-8501, Japan

*Correspondence should be addressed to: Wataru Aoki

Tel.: +81-75-753-6495; Email: aoki.wataru.6a@kyoto-u.ac.jp

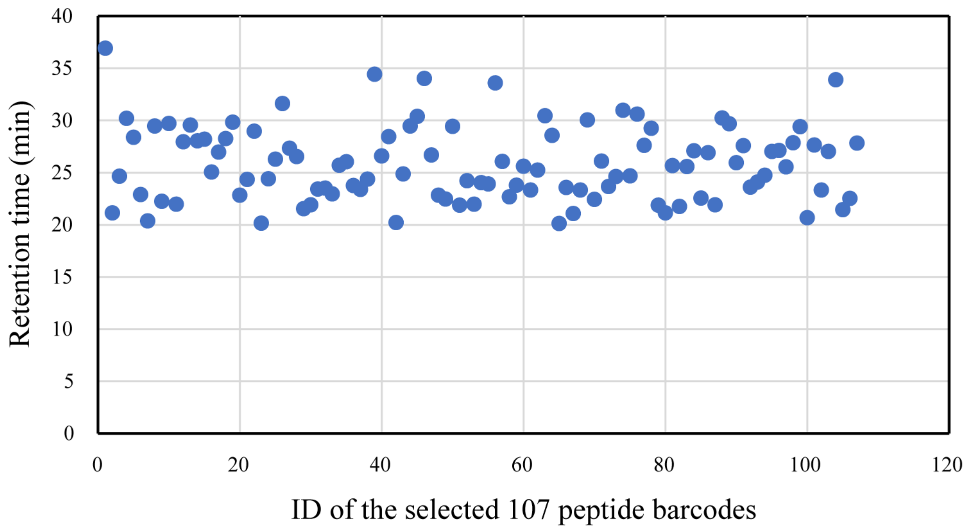

Supplementary Fig. 1. Retention times of the selected peptide barcodes.

Scatter plot of the retention times of the selected peptide barcodes in liquid chromatography–tandem mass spectrometry analysis. This figure was created using Illustrator CS2 (https://www.adobe.com/).

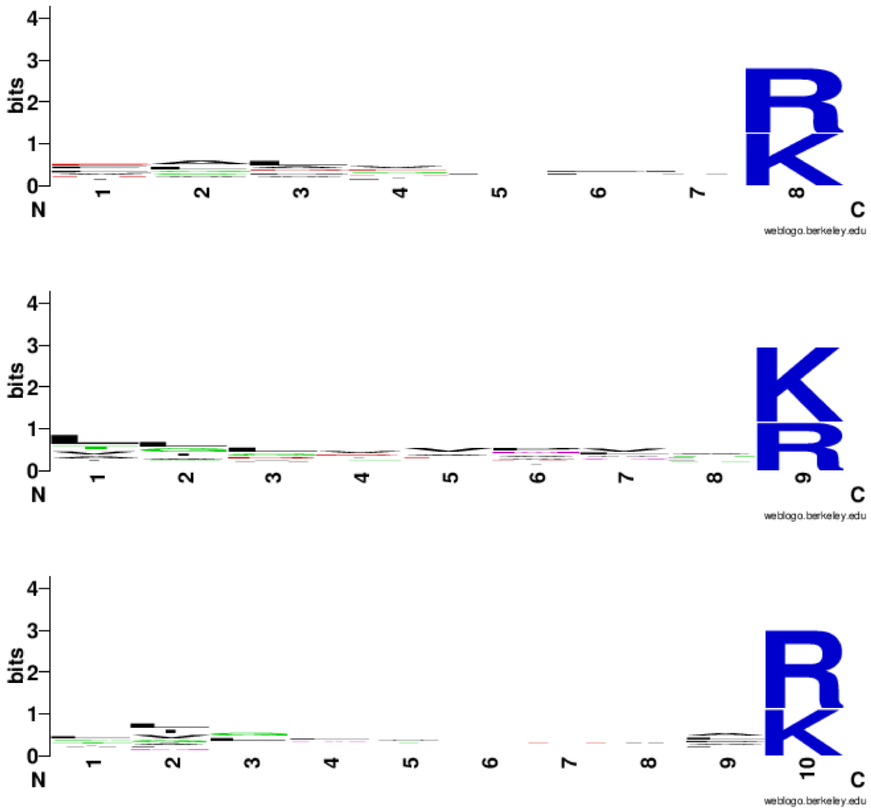

Supplementary Fig. 2. WebLogo sequences of 7-, 8- or 9-amino-acid length of the peptide barcode sequences selected from the yeast SRMAtlas.

We selected 29, 38 and 40 peptides for 8-, 9- or 10-amino-acid-length peptide barcodes. WebLogo sequences were created for peptide barcodes of 8-, 9-, or 10-amino-acid lengths and showed no sequence similarity. This figure was created using Illustrator CS2 (https://www.adobe.com/).

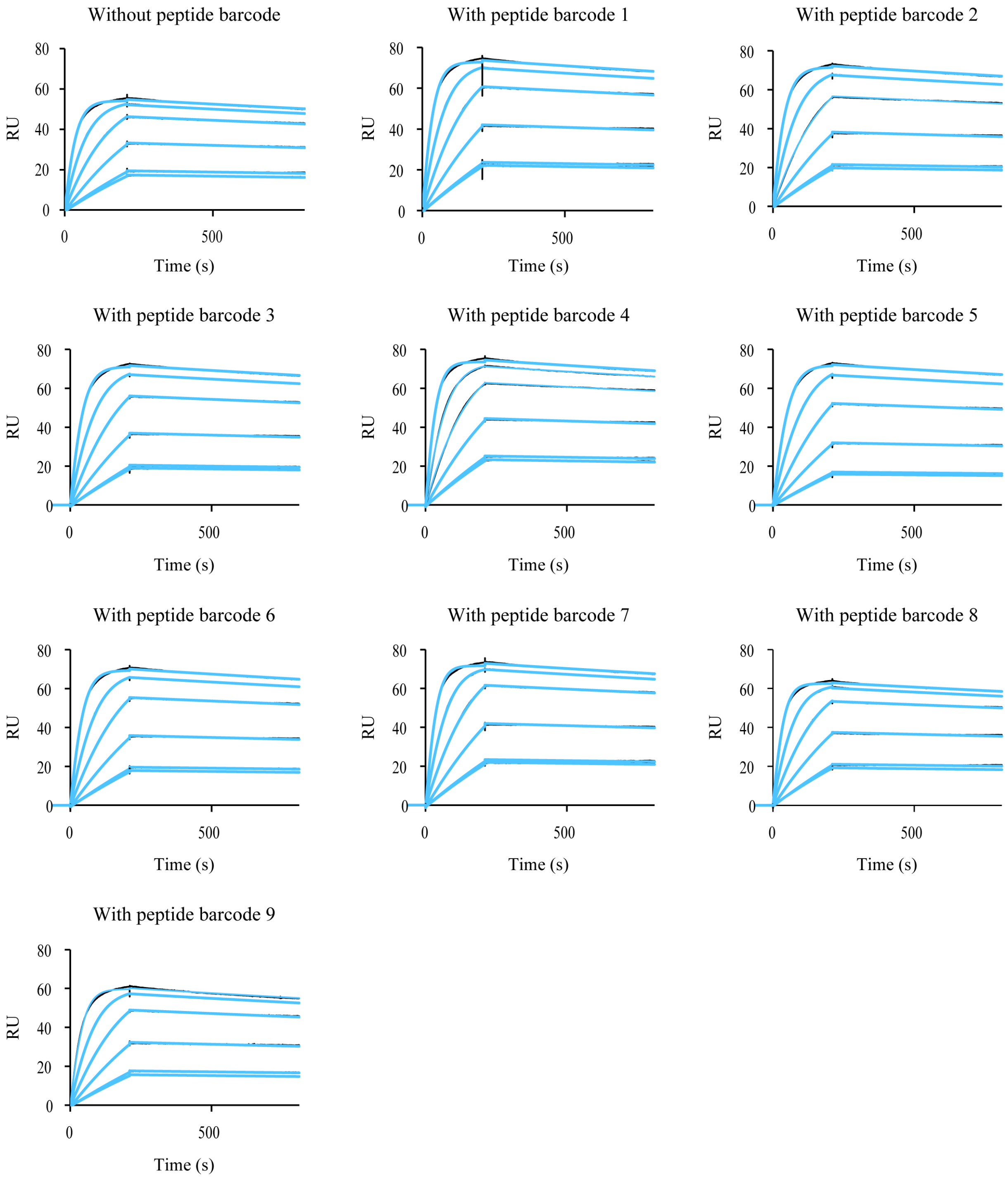

Supplementary Fig. 3. Surface plasmon resonance (SPR) analysis of anti-green fluorescent protein (GFP) wild-type nanobody (Nb) fused with various peptide barcodes shown in Fig. 2c.

SPR analysis was conducted using BIACORE T-200 with a GFP-immobilised CM5 chip. Anti-

GFP Nbs (0.625, 1.25, 2.5, 5 or 10 nM) was subjected to SPR in sequence, and the 0.625 nM analyte was again subjected to SPR to confirm reproducibility. Black lines show raw data, and blue lines show regression curves. This figure was created using Illustrator CS2 (https://www.adobe.com/).

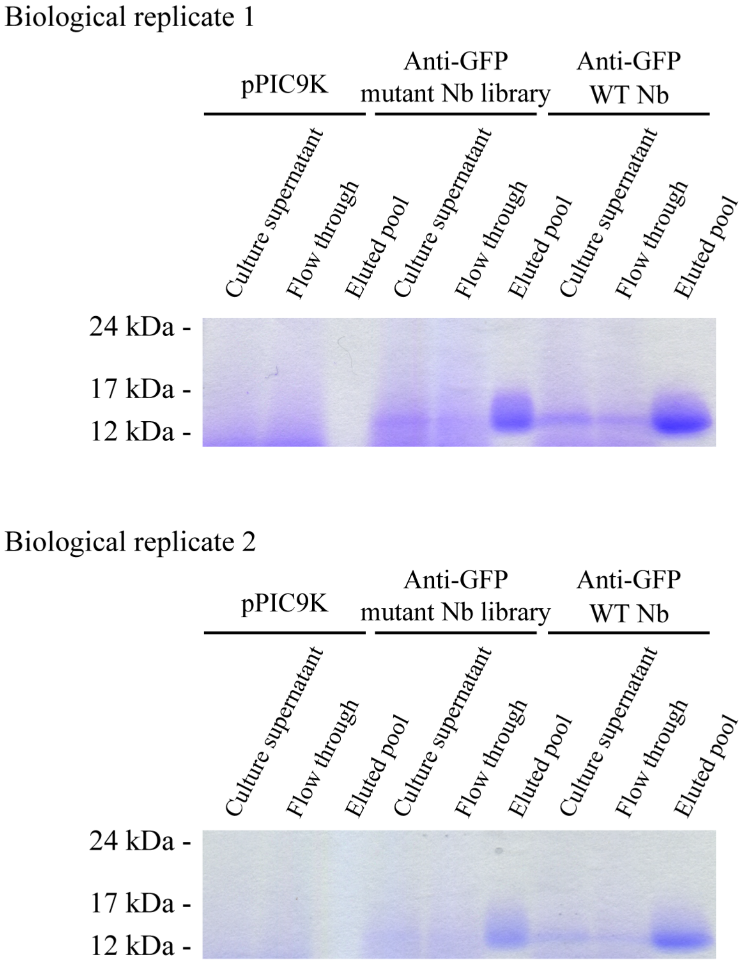

Supplementary Fig. 4. Construction of the anti-green fluorescent protein (GFP) mutant nanobody (Nb) library.

An anti-GFP mutant Nb library was produced in one-pot by *Pichia pastoris* and purified using Ni–nitrilotriacetic acid agarose. *P. pastoris* strains harbouring a backbone vector, pPIC9K, or producing anti-GFP WT Nb (16 kDa) were used as a negative and a positive control, respectively. Purified Nbs (about 16 kDa) were subjected to sodium dodecyl sulphate–polyacrylamide gel electrophoresis and stained with Coomassie Brilliant Blue. These gels are cropped and full-length gels are presented in **Supplementary Figs. 13 and 14**. This figure was created using Illustrator CS2 (https://www.adobe.com/).

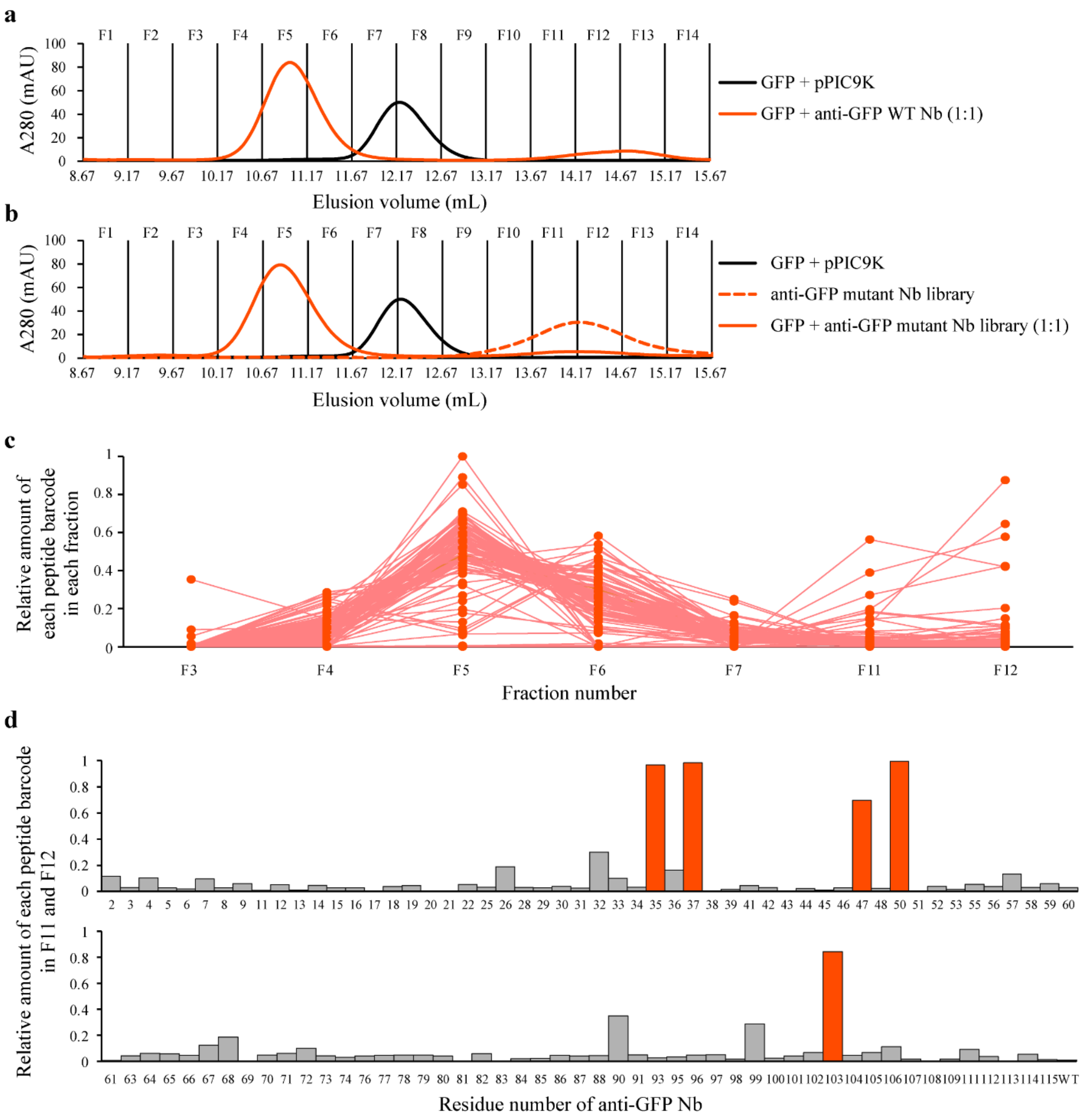

Supplementary Fig. 5. One-pot evaluation of affinities of the anti-green fluorescent protein (GFP) mutant nanobody (Nb) library (the second of two independent experiments)

(a and b) Size-exclusion chromatography (SEC) for separation of functional and nonfunctional Nbs. To confirm the separation of GFP and the GFP–Nb complex, GFP alone or a mixture of equimolar amounts of GFP and anti-GFP wild-type Nb were subjected to SEC in (a). For one-pot evaluation of affinities of the anti-GFP mutant Nb library, GFP alone, the anti-GFP mutant Nb library alone and a mixture of equimolar amounts of GFP and the anti-GFP mutant Nb library

were subjected to SEC in (b). The purified sample from *Pichia pastoris* transformed with a backbone vector (pPIC9K) was used as a control. Fourteen fractions were collected in each experiment. **(c)** Quantification of the relative amount of each peptide barcode in each fraction. The total amount of each peptide barcode in fractions F3–F7 and F11–F12 was defined as 1. Each line indicates each peptide barcode. **(d)** Identification of nonfunctional anti-GFP mutant Nbs. The graph shows the relative amount of each peptide barcode in fractions F11 and F12 in which nonfunctional mutant Nbs were enriched. The total amount of each peptide barcode in fractions F3–F7 and F11–F12 was defined as 1. Five nonfunctional anti-GFP mutant Nbs whose peptide barcodes were mostly detected in fractions F11 and F12 (>50%) are coloured in dark red. Anti-GFP mutant Nbs whose peptide barcodes were not identified by mass spectrometry are not shown. The data shown are the second of two independent experiments, and the first showed equivalent results to the second (**Fig. 3**). This figure was created using Illustrator CS2 (https://www.adobe.com/).

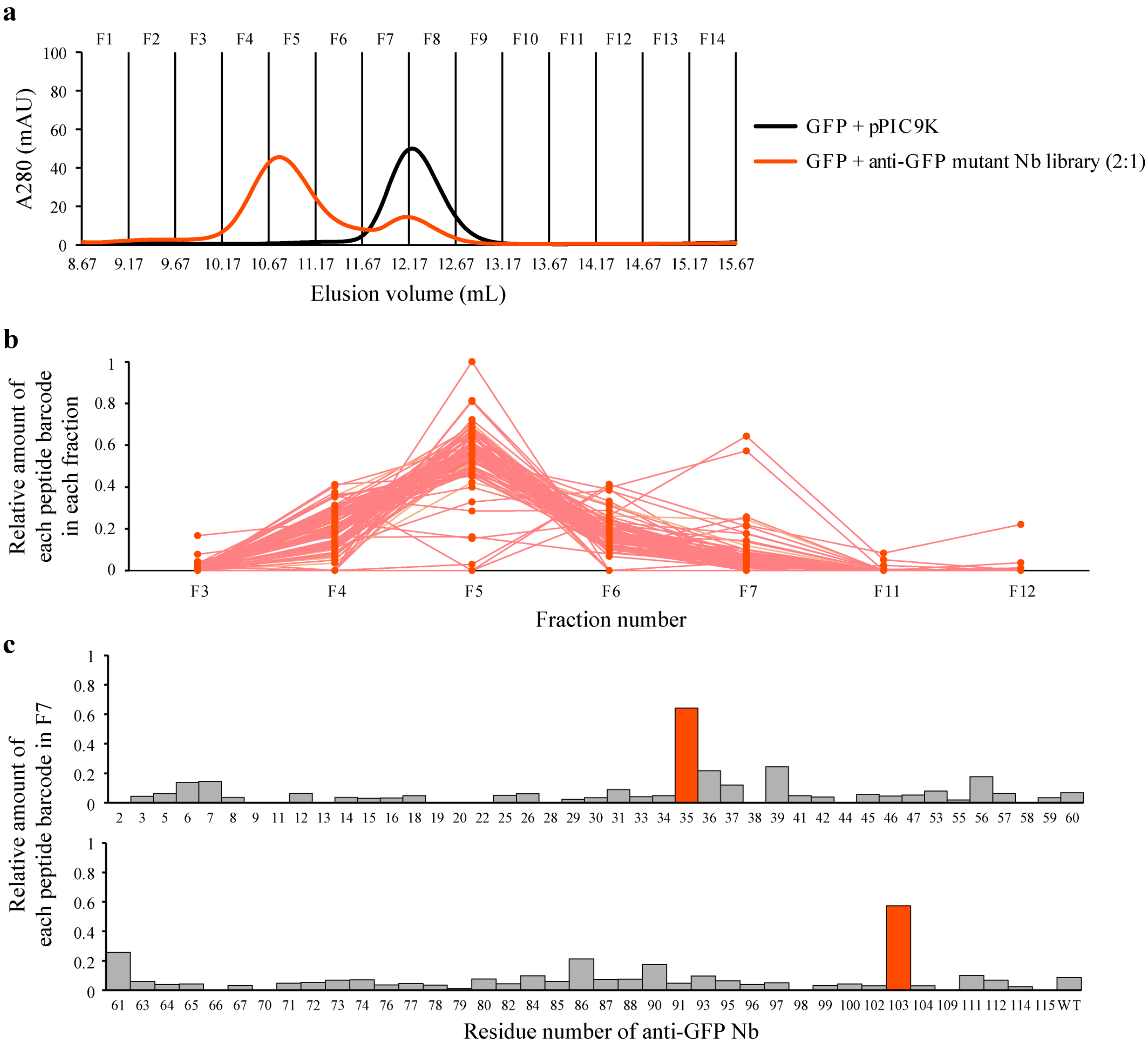

Supplementary Fig. 6. Separation of low-affinity and nonfunctional nanobodies (Nbs) by varying the green fluorescent protein (GFP)/Nb molar ratio (the second of two independent experiments)

(a) Size-exclusion chromatography (SEC) for separation of low-affinity and nonfunctional Nbs. For one-pot evaluation of affinities of the anti-GFP mutant Nb library, GFP alone and a mixture of GFP and the anti-GFP mutant Nb library (2:1 molar amount) were subjected to SEC. The purified sample from *Pichia pastoris* transformed with a backbone vector (pPIC9K) was used as a control. Fourteen fractions were collected in each experiment. **(b)** Quantification of the relative amount of each peptide barcode in each fraction. The total amount of each peptide barcode in

fractions F3–F7 was defined as 1. Each line indicates each peptide barcode. **(c)** Identification of nonfunctional anti-GFP mutant Nbs. The graph shows the relative amount of each peptide barcode in fraction F7 in which nonfunctional mutant Nbs were enriched. The total amount of each peptide barcode in fractions F3–F7 was defined as 1. Two nonfunctional anti-GFP mutant Nbs whose peptide barcodes were mostly detected in fraction F7 (>50%) are coloured in dark red. Anti-GFP mutant Nbs whose peptide barcodes were not identified by mass spectrometry (including G50A) are not shown. The data shown are the second of two independent experiments, and the first showed equivalent results to the second (**Fig. 4**). This figure was created using Illustrator CS2 (https://www.adobe.com/).

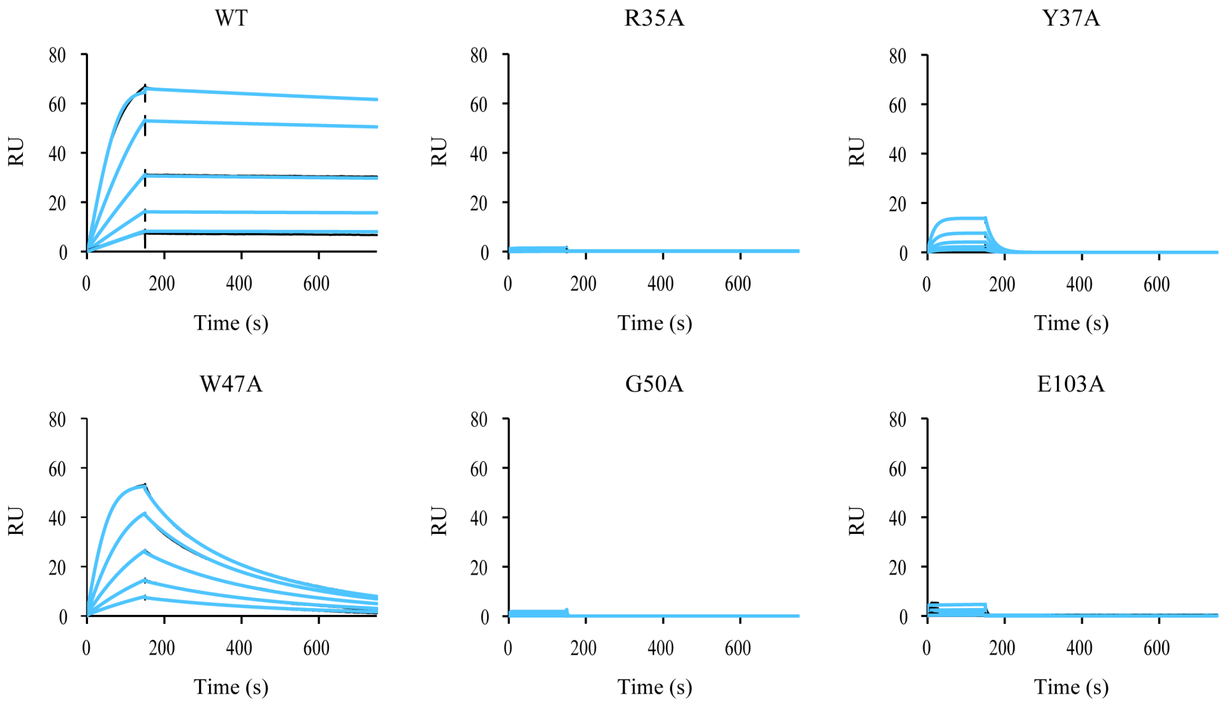

Supplementary Fig. 7. Surface plasmon resonance (SPR) analysis of anti-green fluorescent protein (GFP) mutant nanobodies (Nbs) identified to have decreased affinities by peptide barcoding

SPR analysis was conducted using BIACORE T-200 with a GFP-immobilised CM5 chip. Each anti-GFP mutant Nb analyte (0.3125, 0.625, 1.25, 2.5 or 5 nM) was subjected to SPR in sequence, and the 0.3125 nM analyte was again subjected to SPR to confirm reproducibility. Black lines show raw data, and blue lines show regression curves. This figure was created using Illustrator CS2 (https://www.adobe.com/).

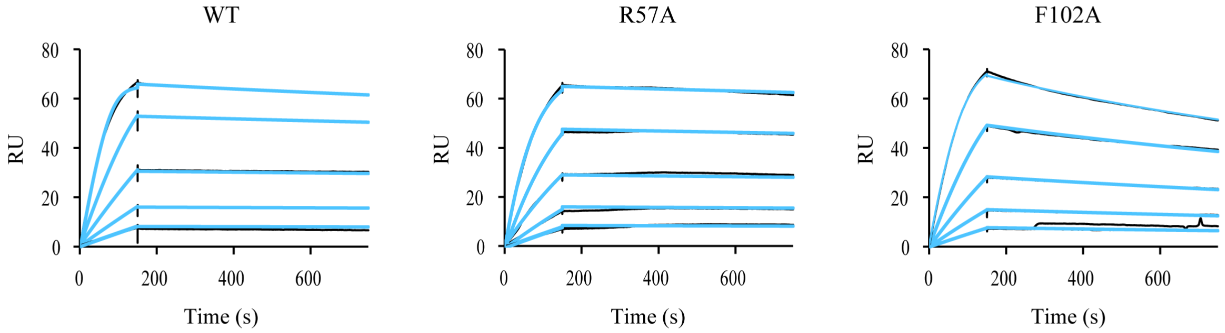

Supplementary Fig. 8. Surface plasmon resonance (SPR) analysis of anti-green fluorescent protein (GFP) mutant nanobodies (Nbs) identified by binding free energy analysis

SPR analysis was conducted using BIACORE T-200 with a GFP-immobilised CM5 chip. Each anti-GFP mutant Nb analyte (0.3125, 0.625, 1.25, 2.5 or 5 nM) was subjected to SPR in sequence, and the 0.3125 nM analyte was again subjected to SPR to confirm reproducibility. Black lines show raw data, and blue lines show regression curves. This figure was created using Illustrator CS2 (https://www.adobe.com/).

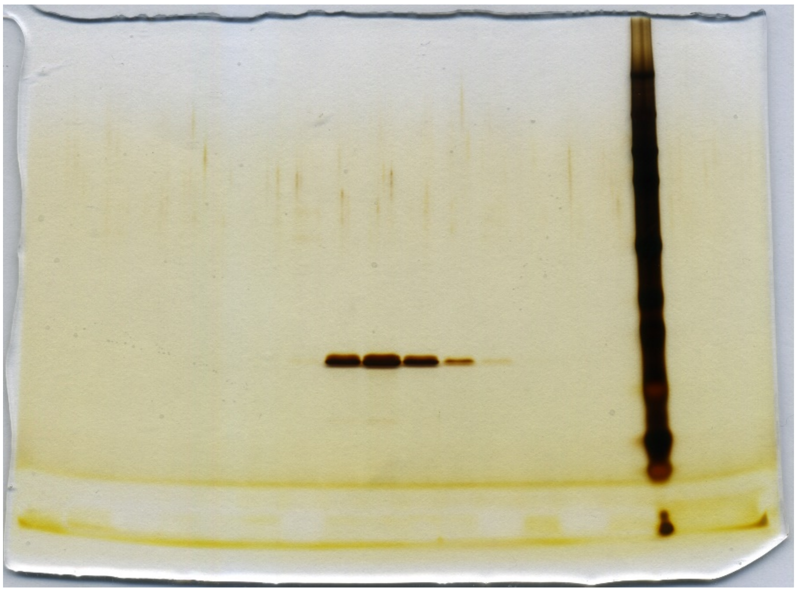

Supplementary Fig. 9. The full-length gel shown in Fig. 3c

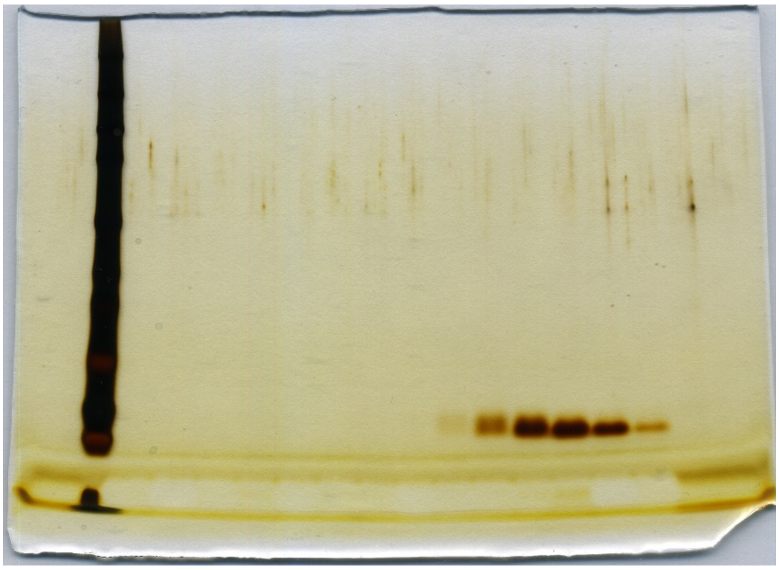

Supplementary Fig. 10. The full-length gel shown in Fig. 3d

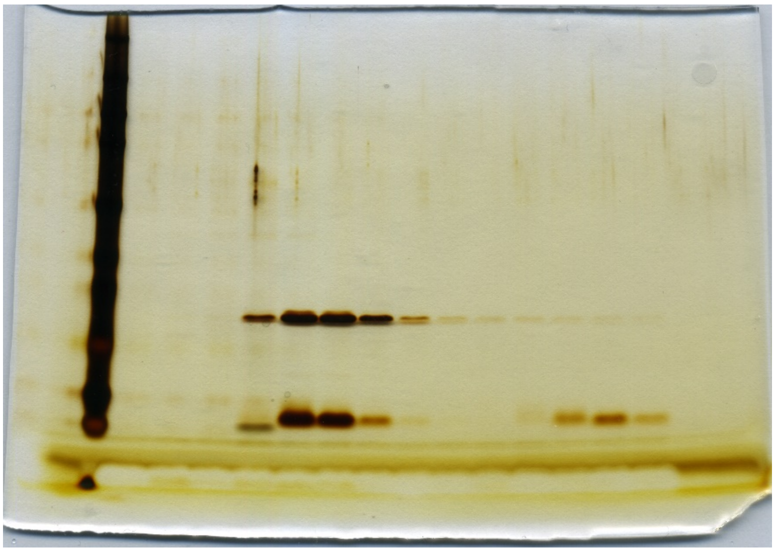

Supplementary Fig. 11. The full-length gel shown in Fig. 3e

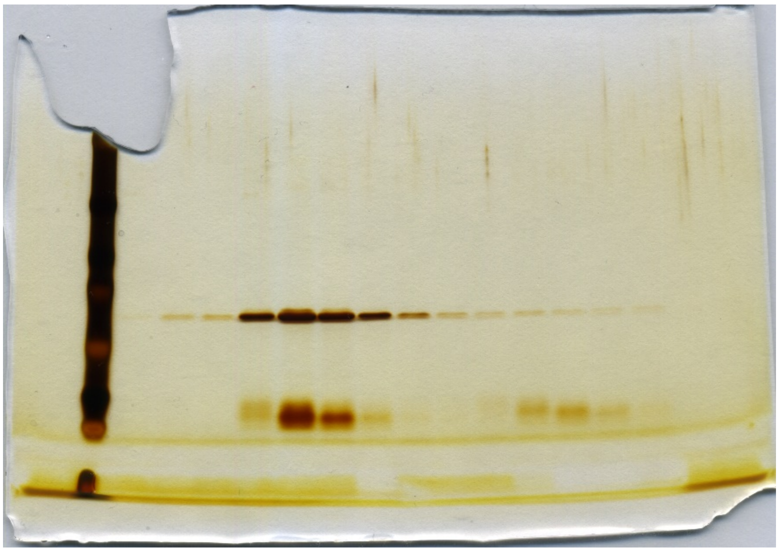

Supplementary Fig. 12. The full-length gel shown in Fig. 3f

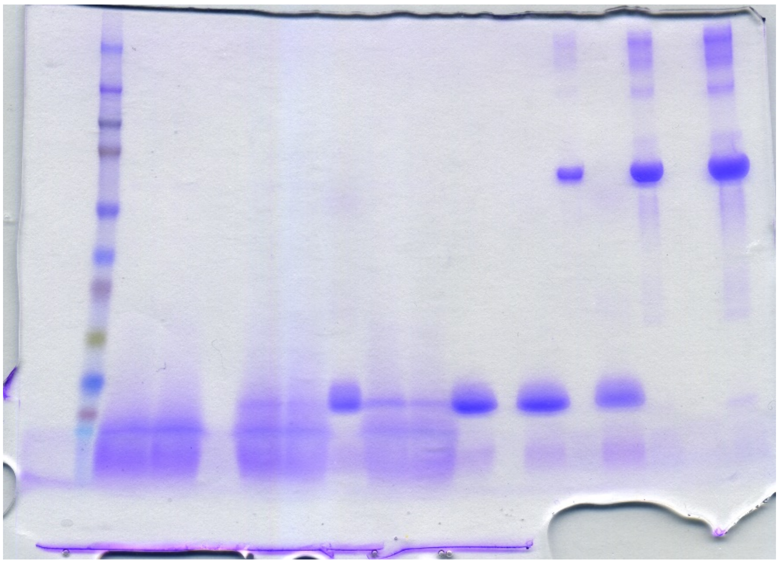

Supplementary Fig. 13. The full-length gel shown in Supplementary Fig. 4 (Biological replicate 1)

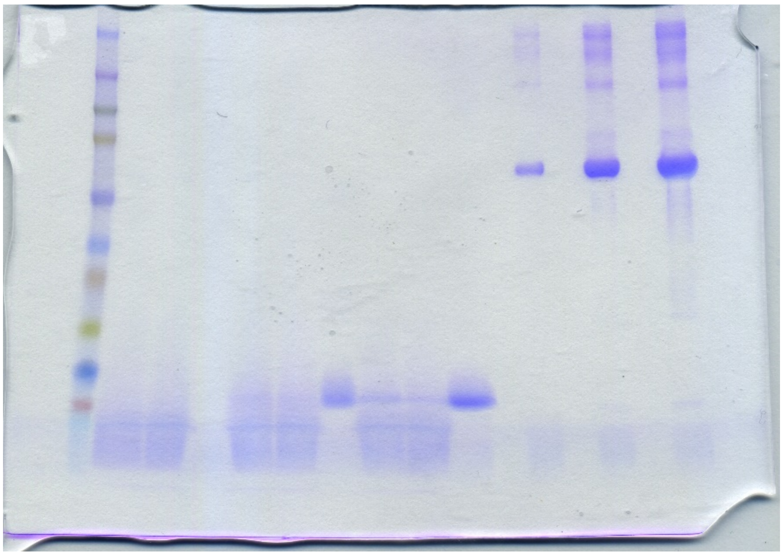

Supplementary Fig. 14. The full-length gel shown in Supplementary Fig. 4 (Biological replicate 2)

227. pPIC9K_6×His (9312 bp)

AGATCTAACATCCAAAGACGAAAGGTTGAATGAAACCTTTTTGCCATCCGACATCCACAGGTCCATTCTCACACATAAGTGCCAAACGCAACAGGAGGGGATACACT
TCTAGATTGTAGGTTTCTGCTTTCCAACTTACTTTGGAAAAACGGTAGGCTGTAGGTGTCCAGGTAAGAGTGTGTATTCACGGTTTGCGTTGTCCTCCCCTATGTGA
AOX1 promoter
20 40 60 80 100

AGCAGCAGACCGTTGCAAACGCAGGACCTCCACTCCTCTTCTCCTCAACACCCACTTTTGCCATCGAAAAACCAGCCCAGTTATTGGGCTTGATTGGAGCTCGCTCA
TCGTCGTCTGGCAACGTTTGCGTCCTGGAGGTGAGGAGAAGAGGAGTTGTGGGTGAAAACGGTAGCTTTTTGGTCGGGTCAATAACCCGAACTAACCTCGAGCGAGT
AOX1 promoter
120 140 160 180 200

TTCCAATTCCTTCTATTAGGCTACTAACACCATGACTTTATTAGCCTGTCTATCCTGGCCCCCCTGGCGAGGTTCATGTTTGTTTATTTCCGAATGCAACAAGCTCC
AAGGTTAAGGAAGATAATCCGATGATTGTGGTACTGAAATAATCGGACAGATAGGACCGGGGGGACCGCTCCAAGTACAAACAAATAAAGGCTTACGTTGTTCGAGG
AOX1 promoter
220 240 260 280 300 320

GCATTACACCCGAACATCACTCCAGATGAGGGCTTTCTGAGTGTGGGGTCAAATAGTTTCATGTTCCCCAAATGGCCCAAAACTGACAGTTTAAACGCTGTCTTGGA
CGTAATGTGGGCTTGTAGTGAGGTCTACTCCCGAAAGACTCACACCCCAGTTTATCAAAGTACAAGGGGTTTACCGGGTTTTGACTGTCAAATTTGCGACAGAACCT
AOX1 promoter
340 360 380 400 420

ACCTAATATGACAAAAGCGTGATCTCATCCAAGATGAACTAAGTTTGGTTCGTTGAAATGCTAACGGCCAGTTGGTCAAAAAGAAACTTCCAAAAGTCGCCATACCG
TGGATTATACTGTTTTCGCACTAGAGTAGGTTCTACTTGATTCAAACCAAGCAACTTTACGATTGCCGGTCAACCAGTTTTTCTTTGAAGGTTTTCAGCGGTATGGC
AOX1 promoter
440 460 480 500 520

TTTGTCTTGTTTGGTATTGATTGACGAATGCTCAAAAATAATCTCATTAATGCTTAGCGCAGTCTCTCTATCGCTTCTGAACCCCGGTGCACCTGTGCCGAAACGCA
AAACAGAACAAACCATAACTAACTGCTTACGAGTTTTTATTAGAGTAATTACGAATCGCGTCAGAGAGATAGCGAAGACTTGGGGCCACGTGGACACGGCTTTGCGT
AOX1 promoter
540 560 580 600 620 640

AATGGGGAAACACCCGCTTTTTGGATGATTATGCATTGTCTCCACATTGTATGCTTCCAAGATTCTGGTGGGAATACTGCTGATAGCCTAACGTTCATGATCAAAAT
TTACCCCTTTGTGGGCGAAAAACCTACTAATACGTAACAGAGGTGTAACATACGAAGGTTCTAAGACCACCCTTATGACGACTATCGGATTGCAAGTACTAGTTTTA
AOX1 promoter
660 680 700 720 740

TTAACTGTTCTAACCCCTACTTGACAGCAATATATAAACAGAAGGAAGCTGCCCTGTCTTAAACCTTTTTTTTTATCATCATTATTAGCTTACTTTCATAATTGCGA
AATTGACAAGATTGGGGATGAACTGTCGTTATATATTTGTCTTCCTTCGACGGGACAGAATTTGGAAAAAAAAATAGTAGTAATAATCGAATGAAAGTATTAACGCT
AOX1 promoter
760 780 800 820 840

CTGGTTCCAATTGACAAGCTTTTGATTTTAACGACTTTTAACGACAACTTGAGAAGATCAAAAAACAACTAATTATTCGAAGGATCCAAACGATGAGATTTCCTTCA
GACCAAGGTTAACTGTTCGAAAACTAAAATTGCTGAAAATTGCTGTTGAACTCTTCTAGTTTTTTGTTGATTAATAAGCTTCCTAGGTTTGCTACTCTAAAGGAAGT

AOX1 promoter a-fa...nal

860 880 900 920 940 960

ATTTTTACTGCAGTTTTATTCGCAGCATCCTCCGCATTAGCTGCTCCAGTCAACACTACAACAGAAGATGAAACGGCACAAATTCCGGCTGAAGCTGTCATCGGTTA
TAAAAATGACGTCAAAATAAGCGTCGTAGGAGGCGTAATCGACGAGGTCAGTTGTGATGTTGTCTTCTACTTTGCCGTGTTTAAGGCCGACTTCGACAGTAGCCAAT

a-facter secretion signal

980 1,000 1,020 1,040 1,060

CTCAGATTTAGAAGGGGATTTCGATGTTGCTGTTTTGCCATTTTCCAACAGCACAAATAACGGGTTATTGTTTATAAATACTACTATTGCCAGCATTGCTGCTAAAG
GAGTCTAAATCTTCCCCTAAAGCTACAACGACAAAACGGTAAAAGGTTGTCGTGTTTATTGCCCAATAACAAATATTTATGATGATAACGGTCGTAACGACGATTTC

a-facter secretion signal

1,080 1,100 1,120 1,140 1,160

AAGAAGGGGTATCTCTCGAGAAAAGAGAGGCTGAAGCTTACGTAGAATTCCCTAGATTACTAGTGGAGGTTCTCATCATCATCATCATCATTAAGGCCGCGAATTAA
TTCTTCCCCATAGAGAGCTCTTTTCTCTCCGACTTCGAATGCATCTTAAGGGATCTAATGATCACCTCCAAGAGTAGTAGTAGTAGTAGTAATTCCGGCGCTTAATT

1 2 3 4 1 2 3 4 5 6 7
S G G S H H H H H H *

a-facter secretion signal pPIC9k MCS

1,180 1,200 1,220 1,240 1,260 1,280

TTCGCCTTAGACATGACTGTTCCTCAGTTCAAGTTGGGCACTTACGAGAAGACCGGTCTTGCTAGATTCTAATCAAGAGGATGTCAGAATGCCATTTGCCTGAGAGA
AAGCGGAATCTGTACTGACAAGGAGTCAAGTTCAACCCGTGAATGCTCTTCTGGCCAGAACGATCTAAGATTAGTTCTCCTACAGTCTTACGGTAAACGGACTCTCT

AOX1 terminator

1,300 1,320 1,340 1,360 1,380

TGCAGGCTTCATTTTTGATACTTTTTTATTTGTAACCTATATAGTATAGGATTTTTTTTGTCATTTTGTTTCTTCTCGTACGAGCTTGCTCCTGATCAGCCTATCTC
ACGTCCGAAGTAAAAACTATGAAAAAATAAACATTGGATATATCATATCCTAAAAAAAACAGTAAAACAAAGAAGAGCATGCTCGAACGAGGACTAGTCGGATAGAG

AOX1 terminator

1,400 1,420 1,440 1,460 1,480

GCAGCTGATGAATATCTTGTGGTAGGGGTTTGGGAAAATCATTCGAGTTTGATGTTTTTCTTGGTATTTCCCACTCCTCTTCAGAGTACAGAAGATTAAGTGAGAAG
CGTCGACTACTTATAGAACACCATCCCCAAACCCTTTTAGTAAGCTCAAACTACAAAAAGAACCATAAAGGGTGAGGAGAAGTCTCATGTCTTCTAATTCACTCTTC

AOX1 terminator

1,500 1,520 1,540 1,560 1,580 1,600

TTCGTTTGTGCAAGCTTATCGATAAGCTTTAATGCGGTAGTTTATCACAGTTAAATTGCTAACGCAGTCAGGCACCGTGTATGAAATCTAACAATGCGCTCATCGTC
AAGCAAACACGTTCGAATAGCTATTCGAAATTACGCCATCAAATAGTGTCAATTTAACGATTGCGTCAGTCCGTGGCACATACTTTAGATTGTTACGCGAGTAGCAG

1,620 1,640 1,660 1,680 1,700

ATCCTCGGCACCGTCACCCTGGATGCTGTAGGCATAGGCTTGGTTATGCCGGTACTGCCGGGCCTCTTGCGGGATATCGTCCATTCCGACAGCATCGCCAGTCACTA
TAGGAGCCGTGGCAGTGGGACCTACGACATCCGTATCCGAACCAATACGGCCATGACGGCCCGGAGAACGCCCTATAGCAGGTAAGGCTGTCGTAGCGGTCAGTGAT

1,720 1,740 1,760 1,780 1,800

TGGCGTGCTGCTAGCGCTATATGCGTTGATGCAATTTCTATGCGCACCCGTTCTCGGAGCACTGTCCGACCGCTTTGGCCGCCGCCCAGTCCTGCTCGCTTCGCTAC
ACCGCACGACGATCGCGATATACGCAACTACGTTAAAGATACGCGTGGGCAAGAGCCTCGTGACAGGCTGGCGAAACCGGCGGCGGGTCAGGACGAGCGAAGCGATG

1,820 1,840 1,860 1,880 1,900 1,920

TTGGAGCCACTATCGACTACGCGATCATGGCGACCACACCCGTCCTGTGGATCTATCGAATCTAAATGTAAGTTAAAATCTCTAAATAATTAAATAAGTCCCAGTTT
AACCTCGGTGATAGCTGATGCGCTAGTACCGCTGGTGTGGGCAGGACACCTAGATAGCTTAGATTTACATTCAATTTTAGAGATTTATTAATTTATTCAGGGTCAAA

1,940 1,960 1,980 2,000 2,020

CTCCATACGAACCTTAACAGCATTGCGGTGAGCATCTAGACCTTCAACAGCAGCCAGATCCATCACTGCTTGGCCAATATGTTTCAGTCCCTCAGGAGTTACGTCTT
GAGGTATGCTTGGAATTGTCGTAACGCCACTCGTAGATCTGGAAGTTGTCGTCGGTCTAGGTAGTGACGAACCGGTTATACAAAGTCAGGGAGTCCTCAATGCAGAA

2,040 2,060 2,080 2,100 2,120 2,140

GTGAAGTGATGAACTTCTGGAAGGTTGCAGTGTTAACTCCGCTGTATTGACGGGCATATCCGTACGTTGGCAAAGTGTGGTTGGTACCGGAGGAGTAATCTCCACAA
CACTTCACTACTTGAAGACCTTCCAACGTCACAATTGAGGCGACATAACTGCCCGTATAGGCATGCAACCGTTTCACACCAACCATGGCCTCCTCATTAGAGGTGTT

2,160 2,180 2,200 2,220 2,240

CTCTCTGGAGAGTAGGCACCAACAAACACAGATCCAGCGTGTTGTACTTGATCAACATAAGAAGAAGCATTCTCGATTTGCAGGATCAAGTGTTCAGGAGCGTACTG
GAGAGACCTCTCATCCGTGGTTGTTTGTGTCTAGGTCGCACAACATGAACTAGTTGTATTCTTCTTCGTAAGAGCTAAACGTCCTAGTTCACAAGTCCTCGCATGAC

2,260 2,280 2,300 2,320 2,340

ATTGGACATTTCCAAAGCCTGCTCGTAGGTTGCAACCGATAGGGTTGTAGAGTGTGCAATACACTTGCGTACAATTTCAACCCTTGGCAACTGCACAGCTTGGTTGT
TAACCTGTAAAGGTTTCGGACGAGCATCCAACGTTGGCTATCCCAACATCTCACACGTTATGTGAACGCATGTTAAAGTTGGGAACCGTTGACGTGTCGAACCAACA

2,360 2,380 2,400 2,420 2,440 2,460

GAACAGCATCTTCAATTCTGGCAAGCTCCTTGTCTGTCATATCGACAGCCAACAGAATCACCTGGGAATCAATACCATGTTCAGCTTGAGACAGAAGGTCTGAGGCA
CTTGTCGTAGAAGTTAAGACCGTTCGAGGAACAGACAGTATAGCTGTCGGTTGTCTTAGTGGACCCTTAGTTATGGTACAAGTCGAACTCTGTCTTCCAGACTCCGT

2,480 2,500 2,520 2,540 2,560

ACGAAATCTGGATCAGCGTATTTATCAGCAATAACTAGAACTTCAGAAGGCCCAGCAGGCATGTCAATACTACACAGGGCTGATGTGTCATTTTGAACCATCATCTT
TGCTTTAGACCTAGTCGCATAAATAGTCGTTATTGATCTTGAAGTCTTCCGGGTCGTCCGTACAGTTATGATGTGTCCCGACTACACAGTAAAACTTGGTAGTAGAA

2,580 2,600 2,620 2,640 2,660

GGCAGCAGTAACGAACTGGTTTCCTGGACCAAATATTTTGTCACACTTAGGAACAGTTTCTGTTCCGTAAGCCATAGCAGCTACTGCCTGGGCGCCTCCTGCTAGCA
CCGTCGTCATTGCTTGACCAAAGGACCTGGTTTATAAAACAGTGTGAATCCTTGTCAAAGACAAGGCATTCGGTATCGTCGATGACGGACCCGCGGAGGACGATCGT

2,680 2,700 2,720 2,740 2,760 2,780

CGATACACTTAGCACCAACCTTGTGGGCAACGTAGATGACTTCTGGGGTAAGGGTACCATCCTTCTTAGGTGGAGATGCAAAAACAATTTCTTTGCAACCAGCAACT
GCTATGTGAATCGTGGTTGGAACACCCGTTGCATCTACTGAAGACCCCATTCCCATGGTAGGAAGAATCCACCTCTACGTTTTTGTTAAAGAAACGTTGGTCGTTGA

2,800 2,820 2,840 2,860 2,880

TTGGCAGGAACACCCAGCATCAGGGAAGTGGAAGGCAGAATTGCGGTTCCACCAGGAATATAGAGGCCAACTTTCTCAATAGGTCTTGCAAAACGAGAGCAGACTAC
AACCGTCCTTGTGGGTCGTAGTCCCTTCACCTTCCGTCTTAACGCCAAGGTGGTCCTTATATCTCCGGTTGAAAGAGTTATCCAGAACGTTTTGCTCTCGTCTGATG
2,900 2,920 2,940 2,960 2,980

ACCAGGGCAAGTCTCAACTTGCAACGTCTCCGTTAGTTGAGCTTCATGGAATTTCCTGACGTTATCTATAGAGAGATCAATGGCTCTCTTAACGTTATCTGGCAATT
TGGTCCCGTTCAGAGTTGAACGTTGCAGAGGCAATCAACTCGAAGTACCTTAAAGGACTGCAATAGATATCTCTCTAGTTACCGAGAGAATTGCAATAGACCGTTAA
3,000 3,020 3,040 3,060 3,080 3,100

GCATAAGTTCCTCTGGGAAAGGAGCTTCTAACACAGGTGTCTTCAAAGCGACTCCATCAAACTTGGCAGTTAGTTCTAAAAGGGCTTTGTCACCATTTTGACGAACA
CGTATTCAAGGAGACCCTTTCCTCGAAGATTGTGTCCACAGAAGTTTCGCTGAGGTAGTTTGAACCGTCAATCAAGATTTTCCCGAAACAGTGGTAAAACTGCTTGT
3,120 3,140 3,160 3,180 3,200

TTGTCGACAATTGGTTTGACTAATTCCATAATCTGTTCCGTTTTCTGGATAGGACGACGAAGGGCATCTTCAATTTCTTGTGAGGAGGCCTTAGAAACGTCAATTTT
AACAGCTGTTAACCAAACTGATTAAGGTATTAGACAAGGCAAAAGACCTATCCTGCTGCTTCCCGTAGAAGTTAAAGAACACTCCTCCGGAATCTTTGCAGTTAAAA
3,220 3,240 3,260 3,280 3,300

GCACAATTCAATACGACCTTCAGAAGGGACTTCTTTAGGTTTGGATTCTTCTTTAGGTTGTTCCTTGGTGTATCCTGGCTTGGCATCTCCTTTCCTTCTAGTGACCT
CGTGTTAAGTTATGCTGGAAGTCTTCCCTGAAGAAATCCAAACCTAAGAAGAAATCCAACAAGGAACCACATAGGACCGAACCGTAGAGGAAAGGAAGATCACTGGA
3,320 3,340 3,360 3,380 3,400 3,420

TTAGGGACTTCATATCCAGGTTTCTCTCCACCTCGTCCAACGTCACACCGTACTTGGCACATCTAACTAATGCAAAATAAAATAAGTCAGCACATTCCCAGGCTATA
AATCCCTGAAGTATAGGTCCAAAGAGAGGTGGAGCAGGTTGCAGTGTGGCATGAACCGTGTAGATTGATTACGTTTTATTTTATTCAGTCGTGTAAGGGTCCGATAT
3,440 3,460 3,480 3,500 3,520

TCTTCCTTGGATTTAGCTTCTGCAAGTTCATCAGCTTCCTCCCTAATTTTAGCGTTCAACAAAACTTCGTCGTCAAATAACCGTTTGGTATAAGAACCTTCTGGAGC
AGAAGGAACCTAAATCGAAGACGTTCAAGTAGTCGAAGGAGGGATTAAAATCGCAAGTTGTTTTGAAGCAGCAGTTTATTGGCAAACCATATTCTTGGAAGACCTCG
3,540 3,560 3,580 3,600 3,620

ATTGCTCTTACGATCCCACAAGGTGGCTTCCATGGCTCTAAGACCCTTTGATTGGCCAAAACAGGAAGTGCGTTCCAAGTGACAGAAACCAACACCTGTTTGTTCAA
TAACGAGAATGCTAGGGTGTTCCACCGAAGGTACCGAGATTCTGGGAAACTAACCGGTTTTGTCCTTCACGCAAGGTTCACTGTCTTTGGTTGTGGACAAACAAGTT
3,640 3,660 3,680 3,700 3,720 3,740

CCACAAATTTCAAGCAGTCTCCATCACAATCCAATTCGATACCCAGCAACTTTTGAGTTGCTCCAGATGTAGCACCTTTATACCACAAACCGTGACGACGAGATTGG
GGTGTTTAAAGTTCGTCAGAGGTAGTGTTAGGTTAAGCTATGGGTCGTTGAAAACTCAACGAGGTCTACATCGTGGAAATATGGTGTTTGGCACTGCTGCTCTAACC
3,760 3,780 3,800 3,820 3,840

TAGACTCCAGTTTGTGTCCTTATAGCCTCCGGAATAGACTTTTTGGACGAGTACACCAGGCCCAACGAGTAATTAGAAGAGTCAGCCACCAAAGTAGTGAATAGACC
ATCTGAGGTCAAACACAGGAATATCGGAGGCCTTATCTGAAAAACCTGCTCATGTGGTCCGGGTTGCTCATTAATCTTCTCAGTCGGTGGTTTCATCACTTATCTGG
3,860 3,880 3,900 3,920 3,940

ATCGGGGCGGTCAGTAGTCAAAGACGCCAACAAAATTTCACTGACAGGGAACTTTTTGACATCTTCAGAAAGTTCGTATTCAGTAGTCAATTGCCGAGCATCAATAA
TAGCCCCGCCAGTCATCAGTTTCTGCGGTTGTTTTAAAGTGACTGTCCCTTGAAAAACTGTAGAAGTCTTTCAAGCATAAGTCATCAGTTAACGGCTCGTAGTTATT
3,960 3,980 4,000 4,020 4,040 4,060

TGGGGATTATACCAGAAGCAACAGTGGAAGTCACATCTACCAACTTTGCGGTCTCAGAAAAAGCATAAACAGTTCTACTACCGCCATTAGTGAAACTTTTCAAATCG
ACCCCTAATATGGTCTTCGTTGTCACCTTCAGTGTAGATGGTTGAAACGCCAGAGTCTTTTTCGTATTTGTCAAGATGATGGCGGTAATCACTTTGAAAAGTTTAGC

4,080 4,100 4,120 4,140 4,160

CCCAGTGGAGAAGAAAAAGGCACAGCGATACTAGCATTAGCGGGCAAGGATGCAACTTTATCAACCAGGGTCCTATAGATAACCCTAGCGCCTGGGATCATCCTTTG
GGGTCACCTCTTCTTTTTCCGTGTCGCTATGATCGTAATCGCCCGTTCCTACGTTGAAATAGTTGGTCCCAGGATATCTATTGGGATCGCGGACCCTAGTAGGAAAC

4,180 4,200 4,220 4,240 4,260 4,280

GACAACTCTTTCTGCCAAATCTAGGTCCAAAATCACTTCATTGATACCATTATTGTACAACTTGAGCAAGTTGTCGATCAGCTCCTCAAATTGGTCCTCTGTAACGG
CTGTTGAGAAAGACGGTTTAGATCCAGGTTTTAGTGAAGTAACTATGGTAATAACATGTTGAACTCGTTCAACAGCTAGTCGAGGAGTTTAACCAGGAGACATTGCC

4,300 4,320 4,340 4,360 4,380

ATGACTCAACTTGCACATTAACTTGAAGCTCAGTCGATTGAGTGAACTTGATCAGGTTGTGCAGCTGGTCAGCAGCATAGGGAAACACGGCTTTTCCTACCAAACTC
TACTGAGTTGAACGTGTAATTGAACTTCGAGTCAGCTAACTCACTTGAACTAGTCCAACACGTCGACCAGTCGTCGTATCCCTTTGTGCCGAAAAGGATGGTTTGAG

4,400 4,420 4,440 4,460 4,480

AAGGAATTATCAAACTCTGCAACACTTGCGTATGCAGGTAGCAAGGGAAATGTCATACTTGAAGTCGGACAGTGAGTGTAGTCTTGAGAAATTCTGAAGCCGTATTT
TTCCTTAATAGTTTGAGACGTTGTGAACGCATACGTCCATCGTTCCCTTTACAGTATGAACTTCAGCCTGTCACTCACATCAGAACTCTTTAAGACTTCGGCATAAA

4,500 4,520 4,540 4,560 4,580 4,600

TTATTATCAGTGAGTCAGTCATCAGGAGATCCTCTACGCCGGACGCATCGTGGCCGACCTGCAGGGGGGGGGGGGGGCGCTGAGGTCTGCCTCGTGAAGAAGGTGTTG
AATAATAGTCACTCAGTCAGTAGTCCTCTAGGAGATGCGGCCTGCGTAGCACCGGCTGGACGTCCCCCCCCCCCCCCGCGACTCCAGACGGAGCACTTCTTCCACAAC

4,620 4,640 4,660 4,680 4,700

CTGACTCATACCAGGCCTGAATCGCCCCATCATCCAGCCAGAAAGTGAGGGAGCCACGGTTGATGAGAGCTTTGTTGTAGGTGGACCAGTTGGTGATTTTGAACTTT
GACTGAGTATGGTCCGGACTTAGCGGGGTAGTAGGTCGGTCTTTCACTCCCTCGGTGCCAACTACTCTCGAAACAACATCCACCTGGTCAACCACTAAAACTTGAAA

4,720 4,740 4,760 4,780 4,800

TGCTTTGCCACGGAACGGTCTGCGTTGTCGGGAAGATGCGTGATCTGATCCTTCAACTCAGCAAAAGTTCGATTTATTCAACAAAGCCGCCGTCCCGTCAAGTCAGC
ACGAAACGGTGCCTTGCCAGACGCAACAGCCCTTCTACGCACTAGACTAGGAAGTTGAGTCGTTTTCAAGCTAAATAAGTTGTTTCGGCGGCAGGGCAGTTCAGTCG

4,820 4,840 4,860 4,880 4,900 4,920

GTAATGCTCTGCCAGTGTTACAACCAATTAACCAATTCTGATTAGAAAAACTCATCGAGCATCAAATGAAACTGCAATTTATTCATATCAGGATTATCAATACCATA
CATTACGAGACGGTCACAATGTTGGTTAATTGGTTAAGACTAATCTTTTTGAGTAGCTCGTAGTTTACTTTGACGTTAAATAAGTATAGTCCTAATAGTTATGGTAT

4,940 4,960 4,980 5,000 5,020

TTTTTGAAAAAGCCGTTTCTGTAATGAAGGAGAAAACTCACCGAGGCAGTTCCATAGGATGGCAAGATCCTGGTATCGGTCTGCGATTCCGACTCGTCCAACATCAA
AAAAACTTTTTCGGCAAAGACATTACTTCCTCTTTTGAGTGGCTCCGTCAAGGTATCCTACCGTTCTAGGACCATAGCCAGACGCTAAGGCTGAGCAGGTTGTAGTT

5,040 5,060 5,080 5,100 5,120

TACAACCTATTAATTTCCCCTCGTCAAAAATAAGGTTATCAAGTGAGAAATCACCATGAGTGACGACTGAATCCGGTGAGAATGGCAAAAGCTTATGCATTTCTTTC
ATGTTGGATAATTAAAGGGGAGCAGTTTTTATTCCAATAGTTCACTCTTTAGTGGTACTCACTGCTGACTTAGGCCACTCTTACCGTTTTCGAATACGTAAAGAAAG

5,140 5,160 5,180 5,200 5,220 5,240

CAGACTTGTTCAACAGGCCAGCCATTACGCTCGTCATCAAAATCACTCGCATCAACCAAACCGTTATTCATTCGTGATTGCGCCTGAGCGAGACGAAATACGCGATC
GTCTGAACAAGTTGTCCGGTCGGTAATGCGAGCAGTAGTTTTAGTGAGCGTAGTTGGTTTGGCAATAAGTAAGCACTAACGCGGACTCGCTCTGCTTTATGCGCTAG
5,260 5,280 5,300 5,320 5,340

GCTGTTAAAAGGACAATTACAAACAGGAATCGAATGCAACCGGCGCAGGAACACTGCCAGCGCATCAACAATATTTTCACCTGAATCAGGATATTCTTCTAATACCT
CGACAATTTTCCTGTTAATGTTTGTCCTTAGCTTACGTTGGCCGCGTCCTTGTGACGGTCGCGTAGTTGTTATAAAAGTGGACTTAGTCCTATAAGAAGATTATGGA
5,360 5,380 5,400 5,420 5,440

GGAATGCTGTTTTCCCGGGGATCGCAGTGGTGAGTAACCATGCATCATCAGGAGTACGGATAAAATGCTTGATGGTCGGAAGAGGCATAAATTCCGTCAGCCAGTTT
CCTTACGACAAAAGGGCCCCTAGCGTCACCACTCATTGGTACGTAGTAGTCCTCATGCCTATTTTACGAACTACCAGCCTTCTCCGTATTTAAGGCAGTCGGTCAAA
5,460 5,480 5,500 5,520 5,540 5,560

AGTCTGACCATCTCATCTGTAACATCATTGGCAACGCTACCTTTGCCATGTTTCAGAAACAACTCTGGCGCATCGGGCTTCCCATACAATCGATAGATTGTCGCACC
TCAGACTGGTAGAGTAGACATTGTAGTAACCGTTGCGATGGAAACGGTACAAAGTCTTTGTTGAGACCGCGTAGCCCGAAGGGTATGTTAGCTATCTAACAGCGTGG
5,580 5,600 5,620 5,640 5,660

TGATTGCCCGACATTATCGCGAGCCCATTTATACCCATATAAATCAGCATCCATGTTGGAATTTAATCGCGGCCTCGAGCAAGACGTTTCCCGTTGAATATGGCTCA
ACTAACGGGCTGTAATAGCGCTCGGGTAAATATGGGTATATTTAGTCGTAGGTACAACCTTAAATTAGCGCCGGAGCTCGTTCTGCAAAGGGCAACTTATACCGAGT
5,680 5,700 5,720 5,740 5,760

TAACACCCCTTGTATTACTGTTTATGTAAGCAGACAGTTTTATTGTTCATGATGATATATTTTTATCTTGTGCAATGTAACATCAGAGATTTTGAGACACAACGTGG
ATTGTGGGGAACATAATGACAAATACATTCGTCTGTCAAAATAACAAGTACTACTATATAAAAATAGAACACGTTACATTGTAGTCTCTAAAACTCTGTGTTGCACC
5,780 5,800 5,820 5,840 5,860 5,880

CTTTCCCCCCCCCCCCCTGCAGGTCGGCATCACCGGCGCCACAGGTGCGGTTGCTGGCGCCTATATCGCCGACATCACCGATGGGGAAGATCGGGCTCGCCACTTCGG
GAAAGGGGGGGGGGGGGACGTCCAGCCGTAGTGGCCGCGGTGTCCACGCCAACGACCGCGGATATAGCGGCTGTAGTGGCTACCCCTTCTAGCCCGAGCGGTGAAGCC
5,900 5,920 5,940 5,960 5,980

GCTCATGAGCGCTTGTTTCGGCGTGGGTATGGTGGCAGGCCCCGTGGCCGGGGGACTGTTGGGCGCCATCTCCTTGCATGCACCATTCCTTGCGGCGGCGGTGCTCA
CGAGTACTCGCGAACAAAGCCGCACCCATACCACCGTCCGGGGCACCGGCCCCCTGACAACCCGCGGTAGAGGAACGTACGTGGTAAGGAACGCCGCCGCCACGAGT
6,000 6,020 6,040 6,060 6,080

ACGGCCTCAACCTACTACTGGGCTGCTTCCTAATGCAGGAGTCGCATAAGGGAGAGCGTCGAGTATCTATGATTGGAAGTATGGGAATGGTGATACCCGCATTCTTC
TGCCGGAGTTGGATGATGACCCGACGAAGGATTACGTCCTCAGCGTATTCCCTCTCGCAGCTCATAGATACTAACCTTCATACCCTTACCACTATGGGCGTAAGAAG
6,100 6,120 6,140 6,160 6,180 6,200

AGTGTCTTGAGGTCTCCTATCAGATTATGCCCAACTAAAGCAACCGGAGGAGGAGATTTCATGGTAAATTTCTCTGACTTTTGGTCATCAGTAGACTCGAACTGTGA
TCACAGAACTCCAGAGGATAGTCTAATACGGGTTGATTTCGTTGGCCTCCTCCTCTAAAGTACCATTTAAAGAGACTGAAAACCAGTAGTCATCTGAGCTTGACACT
6,220 6,240 6,260 6,280 6,300

GACTATCTCGGTTATGACAGCAGAAATGTCCTTCTTGGAGACAGTAAATGAAGTCCCACCAATAAAGAAATCCTTGTTATCAGGAACAAACTTCTTGTTTCGAACTT
CTGATAGAGCCAATACTGTCGTCTTTACAGGAAGAACCTCTGTCATTTACTTCAGGGTGGTTATTTCTTTAGGAACAATAGTCCTTGTTTGAAGAACAAAGCTTGAA
6,320 6,340 6,360 6,380 6,400 6,420

TTTCGGTGCCTTGAACTATAAAATGTAGAGTGGATATGTCGGGTAGGAATGGAGCGGGCAAATGCTTACCTTCTGGACCTTCAAGAGGTATGTAGGGTTTGTAGATA
AAAGCCACGGAACTTGATATTTTACATCTCACCTATACAGCCCATCCTTACCTCGCCCGTTTACGAATGGAAGACCTGGAAGTTCTCCATACATCCCAAACATCTAT

6,440 6,460 6,480 6,500 6,520

CTGATGCCAACTTCAGTGACAACGTTGCTATTTCGTTCAAACCATTCCGAATCCAGAGAAATCAAAGTTGTTTGTCTACTATTGATCCAAGCCAGTGCGGTCTTGAA
GACTACGGTTGAAGTCACTGTTGCAACGATAAAGCAAGTTTGGTAAGGCTTAGGTCTCTTTAGTTTCAACAAACAGATGATAACTAGGTTCGGTCACGCCAGAACTT

6,540 6,560 6,580 6,600 6,620

ACTGACAATAGTGTGCTCGTGTTTTGAGGTCATCTTTGTATGAATAAATCTAGTCTTTGATCTAAATAATCTTGACGAGCCAAGGCGATAAATACCCAAATCTAAAA
TGACTGTTATCACACGAGCACAAAACTCCAGTAGAAACATACTTATTTAGATCAGAAACTAGATTTATTAGAACTGCTCGGTTCCGCTATTTATGGGTTTAGATTTT

6,640 6,660 6,680 6,700 6,720 6,740

CTCTTTTAAAACGTTAAAAGGACAAGTATGTCTGCCTGTATTAAACCCCAAATCAGCTCGTAGTCTGATCCTCATCAACTTGAGGGGCACTATCTTGTTTTAGAGAA
GAGAAAATTTTGCAATTTTCCTGTTCATACAGACGGACATAATTTGGGGTTTAGTCGAGCATCAGACTAGGAGTAGTTGAACTCCCCGTGATAGAACAAAATCTCTT

6,760 6,780 6,800 6,820 6,840

ATTTGCGGAGATGCGATATCGAGAAAAAGGTACGCTGATTTTAAACGTGAAATTTATCTCAAGATCTCTGCCTCGCGCGTTTCGGTGATGACGGTGAAAACCTCTGA
TAAACGCCTCTACGCTATAGCTCTTTTTCCATGCGACTAAAATTTGCACTTTAAATAGAGTTCTAGAGACGGAGCGCGCAAAGCCACTACTGCCACTTTTGGAGACT

6,860 6,880 6,900 6,920 6,940

CACATGCAGCTCCCGGAGACGGTCACAGCTTGTCTGTAAGCGGATGCCGGGAGCAGACAAGCCCGTCAGGGCGCGTCAGCGGGTGTTGGCGGGTGTCGGGGCGCAGC
GTGTACGTCGAGGGCCTCTGCCAGTGTCGAACAGACATTCGCCTACGGCCCTCGTCTGTTCGGGCAGTCCCGCGCAGTCGCCCACAACCGCCCACAGCCCCGCGTCG

6,960 6,980 7,000 7,020 7,040 7,060

NdeI

CATGACCCAGTCACGTAGCGATAGCGGAGTGTATACTGGCTTAACTATGCGGCATCAGAGCAGATTGTACTGAGAGTGCACCATATGCGGTGTGAAATACCGCACAG
GTACTGGGTCAGTGCATCGCTATCGCCTCACATATGACCGAATTGATACGCCGTAGTCTCGTCTAACATGACTCTCACGTGGTATACGCCACACTTTATGGCGTGTC

7,080 7,100 7,120 7,140 7,160

ATGCGTAAGGAGAAAATACCGCATCAGGCGCTCTTCCGCTTCCTCGCTCACTGACTCGCTGCGCTCGGTCGTTCGGCTGCGGCGAGCGGTATCAGCTCACTCAAAGG
TACGCATTCCTCTTTTATGGCGTAGTCCGCGAGAAGGCGAAGGAGCGAGTGACTGAGCGACGCGAGCCAGCAAGCCGACGCCGCTCGCCATAGTCGAGTGAGTTTCC

7,180 7,200 7,220 7,240 7,260

CGGTAATACGGTTATCCACAGAATCAGGGGATAACGCAGGAAAGAACATGTGAGCAAAAGGCCAGCAAAAGGCCAGGAACCGTAAAAAGGCCGCGTTGCTGGCGTTT
GCCATTATGCCAATAGGTGTCTTAGTCCCCTATTGCGTCCTTTCTTGTACACTCGTTTTCCGGTCGTTTTCCGGTCCTTGGCATTTTTCCGGCGCAACGACCGCAAA

ColE1 origin

7,280 7,300 7,320 7,340 7,360 7,380

TTCCATAGGCTCCGCCCCCCTGACGAGCATCACAAAAATCGACGCTCAAGTCAGAGGTGGCGAAACCCGACAGGACTATAAAGATACCAGGCGTTTCCCCCTGGAAG
AAGGTATCCGAGGCGGGGGGACTGCTCGTAGTGTTTTTAGCTGCGAGTTCAGTCTCCACCGCTTTGGGCTGTCCTGATATTTCTATGGTCCGCAAAGGGGGACCTTC

ColE1 origin

7,400 7,420 7,440 7,460 7,480

CTCCCTCGTGCGCTCTCCTGTTCCGACCCTGCCGCTTACCGGATACCTGTCCGCCTTTCTCCCTTCGGGAAGCGTGGCGCTTTCTCAATGCTCACGCTGTAGGTATC
GAGGGAGCACGCGAGAGGACAAGGCTGGGACGGCGAATGGCCTATGGACAGGCGGAAAGAGGGAAGCCCTTCGCACCGCGAAAGAGTTACGAGTGCGACATCCATAG
ColE1 origin
7,500 7,520 7,540 7,560 7,580

TCAGTTCGGTGTAGGTCGTTCGCTCCAAGCTGGGCTGTGTGCACGAACCCCCCGTTCAGCCCGACCGCTGCGCCTTATCCGGTAACTATCGTCTTGAGTCCAACCCG
AGTCAAGCCACATCCAGCAAGCGAGGTTCGACCCGACACACGTGCTTGGGGGGCAAGTCGGGCTGGCGACGCGGAATAGGCCATTGATAGCAGAACTCAGGTTGGGC
ColE1 origin
7,600 7,620 7,640 7,660 7,680 7,700

GTAAGACACGACTTATCGCCACTGGCAGCAGCCACTGGTAACAGGATTAGCAGAGCGAGGTATGTAGGCGGTGCTACAGAGTTCTTGAAGTGGTGGCCTAACTACGG
CATTCTGTGCTGAATAGCGGTGACCGTCGTCGGTGACCATTGTCCTAATCGTCTCGCTCCATACATCCGCCACGATGTCTCAAGAACTTCACCACCGGATTGATGCC
ColE1 origin
7,720 7,740 7,760 7,780 7,800

CTACACTAGAAGGACAGTATTTGGTATCTGCGCTCTGCTGAAGCCAGTTACCTTCGGAAAAAGAGTTGGTAGCTCTTGATCCGGCAAACAAACCACCGCTGGTAGCG
GATGTGATCTTCCTGTCATAAACCATAGACGCGAGACGACTTCGGTCAATGGAAGCCTTTTTCTCAACCATCGAGAACTAGGCCGTTTGTTTGGTGGCGACCATCGC
ColE1 origin
7,820 7,840 7,860 7,880 7,900

GTGGTTTTTTTGTTTGCAAGCAGCAGATTACGCGCAGAAAAAAAGGATCTCAAGAAGATCCTTTGATCTTTTCTACGGGGTCTGACGCTCAGTGGAACGAAAACTCA
CACCAAAAAAACAAACGTTCGTCGTCTAATGCGCGTCTTTTTTTCCTAGAGTTCTTCTAGGAAACTAGAAAAGATGCCCCAGACTGCGAGTCACCTTGCTTTTGAGT
ColE1 origin
7,920 7,940 7,960 7,980 8,000 8,020

CGTTAAGGGATTTTGGTCATGAGATTATCAAAAAGGATCTTCACCTAGATCCTTTTAAATTAAAAATGAAGTTTTAAATCAATCTAAAGTATATATGAGTAAACTTG
GCAATTCCCTAAAACCAGTACTCTAATAGTTTTTCCTAGAAGTGGATCTAGGAAAATTTAATTTTTACTTCAAAATTTAGTTAGATTTCATATATACTCATTTGAAC
ColE1 origin
8,040 8,060 8,080 8,100 8,120

GTCTGACAGTTACCAATGCTTAATCAGTGAGGCACCTATCTCAGCGATCTGTCTATTTCGTTCATCCATAGTTGCCTGACTCCCCGTCGTGTAGATAACTACGATAC
CAGACTGTCAATGGTTACGAATTAGTCACTCCGTGGATAGAGTCGCTAGACAGATAAAGCAAGTAGGTATCAACGGACTGAGGGGCAGCACATCTATTGATGCTATG
AmpR
8,140 8,160 8,180 8,200 8,220

GGGAGGGCTTACCATCTGGCCCCAGTGCTGCAATGATACCGCGAGACCCACGCTCACCGGCTCCAGATTTATCAGCAATAAACCAGCCAGCCGGAAGGGCCGAGCGC
CCCTCCCGAATGGTAGACCGGGGTCACGACGTTACTATGGCGCTCTGGGTGCGAGTGGCCGAGGTCTAAATAGTCGTTATTTGGTCGGTCGGCCTTCCCGGCTCGCG
AmpR
8,240 8,260 8,280 8,300 8,320 8,340

AGAAGTGGTCCTGCAACTTTATCCGCCTCCATCCAGTCTATTAATTGTTGCCGGGAAGCTAGAGTAAGTAGTTCGCCAGTTAATAGTTTGCGCAACGTTGTTGCCAT
TCTTCACCAGGACGTTGAAATAGGCGGAGGTAGGTCAGATAATTAACAACGGCCCTTCGATCTCATTCATCAAGCGGTCAATTATCAAACGCGTTGCAACAACGGTA
AmpR
8,360 8,380 8,400 8,420 8,440

TGCTGCAGGCATCGTGGTGTCACGCTCGTCGTTTGGTATGGCTTCATTCAGCTCCGGTTCCCAACGATCAAGGCGAGTTACATGATCCCCCATGTTGTGCAAAAAAG
ACGACGTCCGTAGCACCACAGTGCGAGCAGCAAACCATACCGAAGTAAGTCGAGGCCAAGGGTTGCTAGTTCCGCTCAATGTACTAGGGGGTACAACACGTTTTTTC
AmpR
8,460 8,480 8,500 8,520 8,540 8,560

CGGTTAGCTCCTTCGGTCCTCCGATCGTTGTCAGAAGTAAGTTGGCCGCAGTGTTATCACTCATGGTTATGGCAGCACTGCATAATTCTCTTACTGTCATGCCATCC
GCCAATCGAGGAAGCCAGGAGGCTAGCAACAGTCTTCATTCAACCGGCGTCACAATAGTGAGTACCAATACCGTCGTGACGTATTAAGAGAATGACAGTACGGTAGG
AmpR
8,580 8,600 8,620 8,640 8,660

GTAAGATGCTTTTCTGTGACTGGTGAGTACTCAACCAAGTCATTCTGAGAATAGTGTATGCGGCGACCGAGTTGCTCTTGCCCGGCGTCAACACGGGATAATACCGC
CATTCTACGAAAAGACACTGACCACTCATGAGTTGGTTCAGTAAGACTCTTATCACATACGCCGCTGGCTCAACGAGAACGGGCCGCAGTTGTGCCCTATTATGGCG
AmpR
8,680 8,700 8,720 8,740 8,760

GCCACATAGCAGAACTTTAAAAGTGCTCATCATTGGAAAACGTTCTTCGGGGCGAAAACTCTCAAGGATCTTACCGCTGTTGAGATCCAGTTCGATGTAACCCACTC
CGGTGTATCGTCTTGAAATTTTCACGAGTAGTAACCTTTTGCAAGAAGCCCCGCTTTTGAGAGTTCCTAGAATGGCGACAACTCTAGGTCAAGCTACATTGGGTGAG
AmpR
8,780 8,800 8,820 8,840 8,860 8,880

GTGCACCCAACTGATCTTCAGCATCTTTTACTTTCACCAGCGTTTCTGGGTGAGCAAAAACAGGAAGGCAAAATGCCGCAAAAAAGGGAATAAGGGCGACACGGAAA
CACGTGGGTTGACTAGAAGTCGTAGAAAATGAAAGTGGTCGCAAAGACCCACTCGTTTTTGTCCTTCCGTTTTACGGCGTTTTTTCCCTTATTCCCGCTGTGCCTTT
8,900 8,920 8,940 8,960 8,980

TGTTGAATACTCATACTCTTCCTTTTTCAATATTATTGAAGCATTTATCAGGGTTATTGTCTCATGAGCGGATACATATTTGAATGTATTTAGAAAAATAAACAAAT
ACAACTTATGAGTATGAGAAGGAAAAAGTTATAATAACTTCGTAAATAGTCCCAATAACAGAGTACTCGCCTATGTATAAACTTACATAAATCTTTTTATTTGTTTA
9,000 9,020 9,040 9,060 9,080

AGGGGTTCCGCGCACATTTCCCCGAAAAGTGCCACCTGACGTCTAAGAAACCATTATTATCATGACATTAACCTATAAAAATAGGCGTATCACGAGGCCCTTTCGTC
TCCCCAAGGCGCGTGTAAAGGGGCTTTTCACGGTGGACTGCAGATTCTTTGGTAATAATAGTACTGTAATTGGATATTTTTATCCGCATAGTGCTCCGGGAAAGCAG
9,100 9,120 9,140 9,160 9,180 9,200

TTCAAGAATTAATTCTCATGTTTGACAGCTTATCATCGATAAGCTGACTCATGTTGGTATTGTGAAATAGACGCAGATCGGGAACACTGAAAAATAACAGTTATTAT
AAGTTCTTAATTAAGAGTACAAACTGTCGAATAGTAGCTATTCGACTGAGTACAACCATAACACTTTATCTGCGTCTAGCCCTTGTGACTTTTTATTGTCAATAATA
9,220 9,240 9,260 9,280 9,300

TCG
AGC
9,310

101. pPIC9K_anti-GFPNb_Q1A (9688 bp)

```
AGATCTAACATCCAAAGACGAAAGGTTGAATGAAACCTTTTTGCCATCCGACATCCACAGGTCCATTCTCACACATAAGTGCCAAACGCAACAGGAGGGGATACACT
TCTAGATTGTAGGTTTCTGCTTTCCAACTTACTTTGGAAAAACGGTAGGCTGTAGGTGTCCAGGTAAGAGTGTGTATTCACGGTTTGCGTTGTCCTCCCCTATGTGA
AOX1 promoter
20 40 60 80 100

AGCAGCAGACCGTTGCAAACGCAGGACCTCCACTCCTCTTCTCCTCAACACCCACTTTTGCCATCGAAAAACCAGCCCAGTTATTGGGCTTGATTGGAGCTCGCTCA
TCGTCGTCTGGCAACGTTTGCGTCCTGGAGGTGAGGAGAAGAGGAGTTGTGGGTGAAAACGGTAGCTTTTTGGTCGGGTCAATAACCCGAACTAACCTCGAGCGAGT
AOX1 promoter
120 140 160 180 200

TTCCAATTCCTTCTATTAGGCTACTAACACCATGACTTTATTAGCCTGTCTATCCTGGCCCCCCTGGCGAGGTTCATGTTTGTTTATTTCCGAATGCAACAAGCTCC
AAGGTTAAGGAAGATAATCCGATGATTGTGGTACTGAAATAATCGGACAGATAGGACCGGGGGGACCGCTCCAAGTACAAACAAATAAAGGCTTACGTTGTTCGAGG
AOX1 promoter
220 240 260 280 300 320

GCATTACACCCGAACATCACTCCAGATGAGGGCTTTCTGAGTGTGGGGTCAAATAGTTTCATGTTCCCCAAATGGCCCAAAACTGACAGTTTAAACGCTGTCTTGGA
CGTAATGTGGGCTTGTAGTGAGGTCTACTCCCGAAAGACTCACACCCCAGTTTATCAAAGTACAAGGGGTTTACCGGGTTTTGACTGTCAAATTTGCGACAGAACCT
AOX1 promoter
340 360 380 400 420

ACCTAATATGACAAAAGCGTGATCTCATCCAAGATGAACTAAGTTTGGTTCGTTGAAATGCTAACGGCCAGTTGGTCAAAAAGAAACTTCCAAAAGTCGCCATACCG
TGGATTATACTGTTTTCGCACTAGAGTAGGTTCTACTTGATTCAAACCAAGCAACTTTACGATTGCCGGTCAACCAGTTTTTCTTTGAAGGTTTTCAGCGGTATGGC
AOX1 promoter
440 460 480 500 520

TTTGTCTTGTTTGGTATTGATTGACGAATGCTCAAAAATAATCTCATTAATGCTTAGCGCAGTCTCTCTATCGCTTCTGAACCCCGGTGCACCTGTGCCGAAACGCA
AAACAGAACAAACCATAACTAACTGCTTACGAGTTTTTATTAGAGTAATTACGAATCGCGTCAGAGAGATAGCGAAGACTTGGGGCCACGTGGACACGGCTTTGCGT
AOX1 promoter
540 560 580 600 620 640

AATGGGGAAACACCCGCTTTTTGGATGATTATGCATTGTCTCCACATTGTATGCTTCCAAGATTCTGGTGGGAATACTGCTGATAGCCTAACGTTCATGATCAAAAT
TTACCCCTTTGTGGGCGAAAAACCTACTAATACGTAACAGAGGTGTAACATACGAAGGTTCTAAGACCACCCTTATGACGACTATCGGATTGCAAGTACTAGTTTTA
AOX1 promoter
660 680 700 720 740

TTAACTGTTCTAACCCCTACTTGACAGCAATATATAAACAGAAGGAAGCTGCCCTGTCTTAAACCTTTTTTTTTATCATCATTATTAGCTTACTTTCATAATTGCGA
AATTGACAAGATTGGGGATGAACTGTCGTTATATATTTGTCTTCCTTCGACGGGACAGAATTTGGAAAAAAAAATAGTAGTAATAATCGAATGAAAGTATTAACGCT
AOX1 promoter
760 780 800 820 840
```

```
CTGGTTCCAATTGACAAGCTTTTGATTTTAACGACTTTTAACGACAACTTGAGAAGATCAAAAAACAACTAATTATTCGAAGGATCCAAACGATGAGATTTCCTTCA
GACCAAGGTTAACTGTTCGAAAACTAAAATTGCTGAAAATTGCTGTTGAACTCTTCTAGTTTTTTGTTGATTAATAAGCTTCCTAGGTTTGCTACTCTAAAGGAAGT
AOX1 promoter                                                                              a-fa...nal
860          880          900          920          940          960

ATTTTTACTGCAGTTTTATTCGCAGCATCCTCCGCATTAGCTGCTCCAGTCAACACTACAACAGAAGATGAAACGGCACAAATTCCGGCTGAAGCTGTCATCGGTTA
TAAAAATGACGTCAAAATAAGCGTCGTAGGAGGCGTAATCGACGAGGTCAGTTGTGATGTTGTCTTCTACTTTGCCGTGTTTAAGGCCGACTTCGACAGTAGCCAAT
a-facter secretion signal
980          1,000          1,020          1,040          1,060

CTCAGATTTAGAAGGGGATTTCGATGTTGCTGTTTTGCCATTTTCCAACAGCACAAATAACGGGTTATTGTTTATAAATACTACTATTGCCAGCATTGCTGCTAAAG
GAGTCTAAATCTTCCCCTAAAGCTACAACGACAAAACGGTAAAAGGTTGTCGTGTTTATTGCCCAATAACAAATATTTATGATGATAACGGTCGTAACGACGATTTC
a-facter secretion signal
1,080          1,100          1,120          1,140          1,160

AAGAAGGGGTATCTCTCGAGAAAAGAGAGGCTGAAGCTTACGTAGAATTCATGGCTGTTCAATTGGTTGAATCTGGTGGTGCCCTTGTTCAACCTGGAGGATCACTA
TTCTTCCCCATAGAGAGCTCTTTTCTCTCCGACTTCGAATGCATCTTAAGTACCGACAAGTTAACCAACTTAGACCACCACGGGAACAAGTTGGACCTCCTAGTGAT
                                                     2    4    6    8    10   12   14   16   18
                                                     A V Q L V E S G G A L V Q P G G S L
a-facter secretion signal                            anti-GFP Nb
1,180          1,200          1,220          1,240          1,260          1,280

AGATTATCCTGTGCTGCCTCCGGATTTCCTGTTAACAGGTACTCCATGAGATGGTATAGACAGGCTCCAGGTAAAGAAAGAGAGTGGGTCGCTGGTATGTCATCTGC
TCTAATAGGACACGACGGAGGCCTAAAGGACAATTGTCCATGAGGTACTCTACCATATCTGTCCGAGGTCCATTTCTTTCTCTCACCCAGCGACCATACAGTAGACG
 20   22   24   26   28   30   32   34   36   38   40   42   44   46   48   50   52   54
R L S C A A S G F P V N R Y S M R W Y R Q A P G K E R E W V A G M S S A
anti-GFP Nb
1,300          1,320          1,340          1,360          1,380

TGGAGATAGATCCTCATACGAGGATTCTGTCAAAGGAAGGTTTACTATTAGTCGTGACGACGCACGTAATACCGTTTATTTGCAAATGAATTCTCTGAAACCAGAAG
ACCTCTATCTAGGAGTATGCTCCTAAGACAGTTTCCTTCCAAATGATAATCAGCACTGCTGCGTGCATTATGGCAAATAAACGTTTACTTAAGAGACTTTGGTCTTC
  56   58   60   62   64   66   68   70   72   74   76   78   80   82   84   86   88
G D R S S Y E D S V K G R F T I S R D D A R N T V Y L Q M N S L K P E
anti-GFP Nb
1,400          1,420          1,440          1,460          1,480

ACACTGCAGTATATTACTGCAACGTCAACGTAGGTTTCGAGTACTGGGGCCAAGGTACACAGGTGACGGTGTCTAGTGGCGGTAGTAAGGAGGCAAACGTGACAGGC
TGTGACGTCATATAATGACGTTGCAGTTGCATCCAAAGCTCATGACCCCGGTTCCATGTGTCCACTGCCACAGATCACCGCCATCATTCCTCCGTTTGCACTGTCCG
90   92   94   96   98   100  102  104  106  108  110  112  114  116  118  120  122  124
D T A V Y Y C N V N V G F E Y W G Q G T Q V T V S S G G S K E A N V T G
anti-GFP Nb                                                        peptid...rcode
1,500          1,520          1,540          1,560          1,580          1,600
```

TTACGTTCTAGTGGAGGTTCTCATCATCATCATCATCATTAAGGCCGCGAATTAATTCGCCTTAGACATGACTGTTCCTCAGTTCAAGTTGGGCACTTACGAGAAGA
AATGCAAGATCACCTCCAAGAGTAGTAGTAGTAGTAGTAATTCCGGCGCTTAATTAAGCGGAATCTGTACTGACAAGGAGTCAAGTTCAACCCGTGAATGCTCTTCT
126 128 130 132 134 136 138
L R S S G G S H H H H H H *
1,620 1,640 1,660 1,680 1,700

CCGGTCTTGCTAGATTCTAATCAAGAGGATGTCAGAATGCCATTTGCCTGAGAGATGCAGGCTTCATTTTTGATACTTTTTTATTTGTAACCTATATAGTATAGGAT
GGCCAGAACGATCTAAGATTAGTTCTCCTACAGTCTTACGGTAAACGGACTCTCTACGTCCGAAGTAAAAACTATGAAAAAATAAACATTGGATATATCATATCCTA
AOX1 terminator
1,720 1,740 1,760 1,780 1,800

TTTTTTTGTCATTTTGTTTCTTCTCGTACGAGCTTGCTCCTGATCAGCCTATCTCGCAGCTGATGAATATCTTGTGGTAGGGGTTTGGGAAAATCATTCGAGTTTGA
AAAAAAACAGTAAAACAAAGAAGAGCATGCTCGAACGAGGACTAGTCGGATAGAGCGTCGACTACTTATAGAACACCATCCCCAAACCCTTTTAGTAAGCTCAAACT
AOX1 terminator
1,820 1,840 1,860 1,880 1,900 1,920

TGTTTTTCTTGGTATTTCCCACTCCTCTTCAGAGTACAGAAGATTAAGTGAGAAGTTCGTTTGTGCAAGCTTATCGATAAGCTTTAATGCGGTAGTTTATCACAGTT
ACAAAAAGAACCATAAAGGGTGAGGAGAAGTCTCATGTCTTCTAATTCACTCTTCAAGCAAACACGTTCGAATAGCTATTCGAAATTACGCCATCAAATAGTGTCAA
AOX1 terminator
1,940 1,960 1,980 2,000 2,020

AAATTGCTAACGCAGTCAGGCACCGTGTATGAAATCTAACAATGCGCTCATCGTCATCCTCGGCACCGTCACCCTGGATGCTGTAGGCATAGGCTTGGTTATGCCGG
TTTAACGATTGCGTCAGTCCGTGGCACATACTTTAGATTGTTACGCGAGTAGCAGTAGGAGCCGTGGCAGTGGGACCTACGACATCCGTATCCGAACCAATACGGCC
2,040 2,060 2,080 2,100 2,120 2,140

TACTGCCGGGCCTCTTGCGGGATATCGTCCATTCCGACAGCATCGCCAGTCACTATGGCGTGCTGCTAGCGCTATATGCGTTGATGCAATTTCTATGCGCACCCGTT
ATGACGGCCCGGAGAACGCCCTATAGCAGGTAAGGCTGTCGTAGCGGTCAGTGATACCGCACGACGATCGCGATATACGCAACTACGTTAAAGATACGCGTGGGCAA
2,160 2,180 2,200 2,220 2,240

CTCGGAGCACTGTCCGACCGCTTTGGCCGCCGCCCAGTCCTGCTCGCTTCGCTACTTGGAGCCACTATCGACTACGCGATCATGGCGACCACACCCGTCCTGTGGAT
GAGCCTCGTGACAGGCTGGCGAAACCGGCGGCGGGTCAGGACGAGCGAAGCGATGAACCTCGGTGATAGCTGATGCGCTAGTACCGCTGGTGTGGGCAGGACACCTA
2,260 2,280 2,300 2,320 2,340

CTATCGAATCTAAATGTAAGTTAAAATCTCTAAATAATTAAATAAGTCCCAGTTTCTCCATACGAACCTTAACAGCATTGCGGTGAGCATCTAGACCTTCAACAGCA
GATAGCTTAGATTTACATTCAATTTTAGAGATTTATTAATTTATTCAGGGTCAAAGAGGTATGCTTGGAATTGTCGTAACGCCACTCGTAGATCTGGAAGTTGTCGT
2,360 2,380 2,400 2,420 2,440 2,460

GCCAGATCCATCACTGCTTGGCCAATATGTTTCAGTCCCTCAGGAGTTACGTCTTGTGAAGTGATGAACTTCTGGAAGGTTGCAGTGTTAACTCCGCTGTATTGACG
CGGTCTAGGTAGTGACGAACCGGTTATACAAAGTCAGGGAGTCCTCAATGCAGAACACTTCACTACTTGAAGACCTTCCAACGTCACAATTGAGGCGACATAACTGC
2,480 2,500 2,520 2,540 2,560

GGCATATCCGTACGTTGGCAAAGTGTGGTTGGTACCGGAGGAGTAATCTCCACAACTCTCTGGAGAGTAGGCACCAACAAACACAGATCCAGCGTGTTGTACTTGAT
CCGTATAGGCATGCAACCGTTTCACACCAACCATGGCCTCCTCATTAGAGGTGTTGAGAGACCTCTCATCCGTGGTTGTTTGTGTCTAGGTCGCACAACATGAACTA

2,580 2,600 2,620 2,640 2,660

CAACATAAGAAGAAGCATTCTCGATTTGCAGGATCAAGTGTTCAGGAGCGTACTGATTGGACATTTCCAAAGCCTGCTCGTAGGTTGCAACCGATAGGGTTGTAGAG
GTTGTATTCTTCTTCGTAAGAGCTAAACGTCCTAGTTCACAAGTCCTCGCATGACTAACCTGTAAAGGTTTCGGACGAGCATCCAACGTTGGCTATCCCAACATCTC

2,680 2,700 2,720 2,740 2,760 2,780

TGTGCAATACACTTGCGTACAATTTCAACCCTTGGCAACTGCACAGCTTGGTTGTGAACAGCATCTTCAATTCTGGCAAGCTCCTTGTCTGTCATATCGACAGCCAA
ACACGTTATGTGAACGCATGTTAAAGTTGGGAACCGTTGACGTGTCGAACCAACACTTGTCGTAGAAGTTAAGACCGTTCGAGGAACAGACAGTATAGCTGTCGGTT

2,800 2,820 2,840 2,860 2,880

CAGAATCACCTGGGAATCAATACCATGTTCAGCTTGAGACAGAAGGTCTGAGGCAACGAAATCTGGATCAGCGTATTTATCAGCAATAACTAGAACTTCAGAAGGCC
GTCTTAGTGGACCCTTAGTTATGGTACAAGTCGAACTCTGTCTTCCAGACTCCGTTGCTTTAGACCTAGTCGCATAAATAGTCGTTATTGATCTTGAAGTCTTCCGG

2,900 2,920 2,940 2,960 2,980

CAGCAGGCATGTCAATACTACACAGGGCTGATGTGTCATTTTGAACCATCATCTTGGCAGCAGTAACGAACTGGTTTCCTGGACCAAATATTTTGTCACACTTAGGA
GTCGTCCGTACAGTTATGATGTGTCCCGACTACACAGTAAAACTTGGTAGTAGAACCGTCGTCATTGCTTGACCAAAGGACCTGGTTTATAAAACAGTGTGAATCCT

3,000 3,020 3,040 3,060 3,080 3,100

ACAGTTTCTGTTCCGTAAGCCATAGCAGCTACTGCCTGGGCGCCTCCTGCTAGCACGATACACTTAGCACCAACCTTGTGGGCAACGTAGATGACTTCTGGGGTAAG
TGTCAAAGACAAGGCATTCGGTATCGTCGATGACGGACCCGCGGAGGACGATCGTGCTATGTGAATCGTGGTTGGAACACCCGTTGCATCTACTGAAGACCCCATTC

3,120 3,140 3,160 3,180 3,200

GGTACCATCCTTCTTAGGTGGAGATGCAAAAACAATTTCTTTGCAACCAGCAACTTTGGCAGGAACACCCAGCATCAGGGAAGTGGAAGGCAGAATTGCGGTTCCAC
CCATGGTAGGAAGAATCCACCTCTACGTTTTTGTTAAAGAAACGTTGGTCGTTGAAACCGTCCTTGTGGGTCGTAGTCCCTTCACCTTCCGTCTTAACGCCAAGGTG

3,220 3,240 3,260 3,280 3,300

CAGGAATATAGAGGCCAACTTTCTCAATAGGTCTTGCAAAACGAGAGCAGACTACACCAGGGCAAGTCTCAACTTGCAACGTCTCCGTTAGTTGAGCTTCATGGAAT
GTCCTTATATCTCCGGTTGAAAGAGTTATCCAGAACGTTTTGCTCTCGTCTGATGTGGTCCCGTTCAGAGTTGAACGTTGCAGAGGCAATCAACTCGAAGTACCTTA

3,320 3,340 3,360 3,380 3,400 3,420

TTCCTGACGTTATCTATAGAGAGATCAATGGCTCTCTTAACGTTATCTGGCAATTGCATAAGTTCCTCTGGGAAAGGAGCTTCTAACACAGGTGTCTTCAAAGCGAC
AAGGACTGCAATAGATATCTCTCTAGTTACCGAGAGAATTGCAATAGACCGTTAACGTATTCAAGGAGACCCTTTCCTCGAAGATTGTGTCCACAGAAGTTTCGCTG

3,440 3,460 3,480 3,500 3,520

TCCATCAAACTTGGCAGTTAGTTCTAAAAGGGCTTTGTCACCATTTTGACGAACATTGTCGACAATTGGTTTGACTAATTCCATAATCTGTTCCGTTTTCTGGATAG
AGGTAGTTTGAACCGTCAATCAAGATTTTCCCGAAACAGTGGTAAAACTGCTTGTAACAGCTGTTAACCAAACTGATTAAGGTATTAGACAAGGCAAAAGACCTATC

3,540 3,560 3,580 3,600 3,620

GACGACGAAGGGCATCTTCAATTTCTTGTGAGGAGGCCTTAGAAACGTCAATTTTGCACAATTCAATACGACCTTCAGAAGGGACTTCTTTAGGTTTGGATTCTTCT
CTGCTGCTTCCCGTAGAAGTTAAAGAACACTCCTCCGGAATCTTTGCAGTTAAAACGTGTTAAGTTATGCTGGAAGTCTTCCCTGAAGAAATCCAAACCTAAGAAGA

3,640 3,660 3,680 3,700 3,720 3,740

TTAGGTTGTTCCTTGGTGTATCCTGGCTTGGCATCTCCTTTCCTTCTAGTGACCTTTAGGGACTTCATATCCAGGTTTCTCTCCACCTCGTCCAACGTCACACCGTA
AATCCAACAAGGAACCACATAGGACCGAACCGTAGAGGAAAGGAAGATCACTGGAAATCCCTGAAGTATAGGTCCAAAGAGAGGTGGAGCAGGTTGCAGTGTGGCAT
3,760 3,780 3,800 3,820 3,840

CTTGGCACATCTAACTAATGCAAAATAAAATAAGTCAGCACATTCCCAGGCTATATCTTCCTTGGATTTAGCTTCTGCAAGTTCATCAGCTTCCTCCCTAATTTTAG
GAACCGTGTAGATTGATTACGTTTTATTTTATTCAGTCGTGTAAGGGTCCGATATAGAAGGAACCTAAATCGAAGACGTTCAAGTAGTCGAAGGAGGGATTAAAATC
3,860 3,880 3,900 3,920 3,940

CGTTCAACAAAACTTCGTCGTCAAATAACCGTTTGGTATAAGAACCTTCTGGAGCATTGCTCTTACGATCCCACAAGGTGGCTTCCATGGCTCTAAGACCCTTTGAT
GCAAGTTGTTTTGAAGCAGCAGTTTATTGGCAAACCATATTCTTGGAAGACCTCGTAACGAGAATGCTAGGGTGTTCCACCGAAGGTACCGAGATTCTGGGAAACTA
3,960 3,980 4,000 4,020 4,040 4,060

TGGCCAAAACAGGAAGTGCGTTCCAAGTGACAGAAACCAACACCTGTTTGTTCAACCACAAATTTCAAGCAGTCTCCATCACAATCCAATTCGATACCCAGCAACTT
ACCGGTTTTGTCCTTCACGCAAGGTTCACTGTCTTTGGTTGTGGACAAACAAGTTGGTGTTTAAAGTTCGTCAGAGGTAGTGTTAGGTTAAGCTATGGGTCGTTGAA
4,080 4,100 4,120 4,140 4,160

TTGAGTTGCTCCAGATGTAGCACCTTTATACCACAAACCGTGACGACGAGATTGGTAGACTCCAGTTTGTGTCCTTATAGCCTCCGGAATAGACTTTTTGGACGAGT
AACTCAACGAGGTCTACATCGTGGAAATATGGTGTTTGGCACTGCTGCTCTAACCATCTGAGGTCAAACACAGGAATATCGGAGGCCTTATCTGAAAAACCTGCTCA
4,180 4,200 4,220 4,240 4,260 4,280

ACACCAGGCCCAACGAGTAATTAGAAGAGTCAGCCACCAAAGTAGTGAATAGACCATCGGGGCGGTCAGTAGTCAAAGACGCCAACAAAATTTCACTGACAGGGAAC
TGTGGTCCGGGTTGCTCATTAATCTTCTCAGTCGGTGGTTTCATCACTTATCTGGTAGCCCCGCCAGTCATCAGTTTCTGCGGTTGTTTTAAAGTGACTGTCCCTTG
4,300 4,320 4,340 4,360 4,380

TTTTTGACATCTTCAGAAAGTTCGTATTCAGTAGTCAATTGCCGAGCATCAATAATGGGGATTATACCAGAAGCAACAGTGGAAGTCACATCTACCAACTTTGCGGT
AAAAACTGTAGAAGTCTTTCAAGCATAAGTCATCAGTTAACGGCTCGTAGTTATTACCCCTAATATGGTCTTCGTTGTCACCTTCAGTGTAGATGGTTGAAACGCCA
4,400 4,420 4,440 4,460 4,480

CTCAGAAAAAGCATAAACAGTTCTACTACCGCCATTAGTGAAACTTTTCAAATCGCCCAGTGGAGAAGAAAAAGGCACAGCGATACTAGCATTAGCGGGCAAGGATG
GAGTCTTTTTCGTATTTGTCAAGATGATGGCGGTAATCACTTTGAAAAGTTTAGCGGGTCACCTCTTCTTTTTCCGTGTCGCTATGATCGTAATCGCCCGTTCCTAC
4,500 4,520 4,540 4,560 4,580 4,600

CAACTTTATCAACCAGGGTCCTATAGATAACCCTAGCGCCTGGGATCATCCTTTGGACAACTCTTTCTGCCAAATCTAGGTCCAAAATCACTTCATTGATACCATTA
GTTGAAATAGTTGGTCCCAGGATATCTATTGGGATCGCGGACCCTAGTAGGAAACCTGTTGAGAAAGACGGTTTAGATCCAGGTTTTAGTGAAGTAACTATGGTAAT
4,620 4,640 4,660 4,680 4,700

TTGTACAACTTGAGCAAGTTGTCGATCAGCTCCTCAAATTGGTCCTCTGTAACGGATGACTCAACTTGCACATTAACTTGAAGCTCAGTCGATTGAGTGAACTTGAT
AACATGTTGAACTCGTTCAACAGCTAGTCGAGGAGTTTAACCAGGAGACATTGCCTACTGAGTTGAACGTGTAATTGAACTTCGAGTCAGCTAACTCACTTGAACTA
4,720 4,740 4,760 4,780 4,800

CAGGTTGTGCAGCTGGTCAGCAGCATAGGGAAACACGGCTTTTCCTACCAAACTCAAGGAATTATCAAACTCTGCAACACTTGCGTATGCAGGTAGCAAGGGAAATG
GTCCAACACGTCGACCAGTCGTCGTATCCCTTTGTGCCGAAAAGGATGGTTTGAGTTCCTTAATAGTTTGAGACGTTGTGAACGCATACGTCCATCGTTCCCTTTAC
4,820 4,840 4,860 4,880 4,900 4,920

TCATACTTGAAGTCGGACAGTGAGTGTAGTCTTGAGAAATTCTGAAGCCGTATTTTTATTATCAGTGAGTCAGTCATCAGGAGATCCTCTACGCCGGACGCATCGTG
AGTATGAACTTCAGCCTGTCACTCACATCAGAACTCTTTAAGACTTCGGCATAAAAATAATAGTCACTCAGTCAGTAGTCCTCTAGGAGATGCGGCCTGCGTAGCAC

4,940 4,960 4,980 5,000 5,020

GCCGACCTGCAGGGGGGGGGGGGGGCGCTGAGGTCTGCCTCGTGAAGAAGGTGTTGCTGACTCATACCAGGCCTGAATCGCCCCATCATCCAGCCAGAAAGTGAGGGA
CGGCTGGACGTCCCCCCCCCCCCCCGCGACTCCAGACGGAGCACTTCTTCCACAACGACTGAGTATGGTCCGGACTTAGCGGGGTAGTAGGTCGGTCTTTCACTCCCT

5,040 5,060 5,080 5,100 5,120

GCCACGGTTGATGAGAGCTTTGTTGTAGGTGGACCAGTTGGTGATTTTGAACTTTTGCTTTGCCACGGAACGGTCTGCGTTGTCGGGAAGATGCGTGATCTGATCCT
CGGTGCCAACTACTCTCGAAACAACATCCACCTGGTCAACCACTAAAACTTGAAAACGAAACGGTGCCTTGCCAGACGCAACAGCCCTTCTACGCACTAGACTAGGA

5,140 5,160 5,180 5,200 5,220 5,240

TCAACTCAGCAAAAGTTCGATTTATTCAACAAAGCCGCCGTCCCGTCAAGTCAGCGTAATGCTCTGCCAGTGTTACAACCAATTAACCAATTCTGATTAGAAAAACT
AGTTGAGTCGTTTTCAAGCTAAATAAGTTGTTTCGGCGGCAGGGCAGTTCAGTCGCATTACGAGACGGTCACAATGTTGGTTAATTGGTTAAGACTAATCTTTTTGA

5,260 5,280 5,300 5,320 5,340

CATCGAGCATCAAATGAAACTGCAATTTATTCATATCAGGATTATCAATACCATATTTTTGAAAAAGCCGTTTCTGTAATGAAGGAGAAAACTCACCGAGGCAGTTC
GTAGCTCGTAGTTTACTTTGACGTTAAATAAGTATAGTCCTAATAGTTATGGTATAAAAACTTTTTCGGCAAAGACATTACTTCCTCTTTTGAGTGGCTCCGTCAAG

5,360 5,380 5,400 5,420 5,440

CATAGGATGGCAAGATCCTGGTATCGGTCTGCGATTCCGACTCGTCCAACATCAATACAACCTATTAATTTCCCCTCGTCAAAAATAAGGTTATCAAGTGAGAAATC
GTATCCTACCGTTCTAGGACCATAGCCAGACGCTAAGGCTGAGCAGGTTGTAGTTATGTTGGATAATTAAAGGGGAGCAGTTTTTATTCCAATAGTTCACTCTTTAG

5,460 5,480 5,500 5,520 5,540 5,560

ACCATGAGTGACGACTGAATCCGGTGAGAATGGCAAAAGCTTATGCATTTCTTTCCAGACTTGTTCAACAGGCCAGCCATTACGCTCGTCATCAAAATCACTCGCAT
TGGTACTCACTGCTGACTTAGGCCACTCTTACCGTTTTCGAATACGTAAAGAAAGGTCTGAACAAGTTGTCCGGTCGGTAATGCGAGCAGTAGTTTTAGTGAGCGTA

5,580 5,600 5,620 5,640 5,660

CAACCAAACCGTTATTCATTCGTGATTGCGCCTGAGCGAGACGAAATACGCGATCGCTGTTAAAAGGACAATTACAAACAGGAATCGAATGCAACCGGCGCAGGAAC
GTTGGTTTGGCAATAAGTAAGCACTAACGCGGACTCGCTCTGCTTTATGCGCTAGCGACAATTTTCCTGTTAATGTTTGTCCTTAGCTTACGTTGGCCGCGTCCTTG

5,680 5,700 5,720 5,740 5,760

ACTGCCAGCGCATCAACAATATTTTCACCTGAATCAGGATATTCTTCTAATACCTGGAATGCTGTTTTCCCGGGGATCGCAGTGGTGAGTAACCATGCATCATCAGG
TGACGGTCGCGTAGTTGTTATAAAAGTGGACTTAGTCCTATAAGAAGATTATGGACCTTACGACAAAAGGGCCCCTAGCGTCACCACTCATTGGTACGTAGTAGTCC

5,780 5,800 5,820 5,840 5,860 5,880

AGTACGGATAAAATGCTTGATGGTCGGAAGAGGCATAAATTCCGTCAGCCAGTTTAGTCTGACCATCTCATCTGTAACATCATTGGCAACGCTACCTTTGCCATGTT
TCATGCCTATTTTACGAACTACCAGCCTTCTCCGTATTTAAGGCAGTCGGTCAAATCAGACTGGTAGAGTAGACATTGTAGTAACCGTTGCGATGGAAACGGTACAA

5,900 5,920 5,940 5,960 5,980

TCAGAAACAACTCTGGCGCATCGGGCTTCCCATACAATCGATAGATTGTCGCACCTGATTGCCCGACATTATCGCGAGCCCATTTATACCCATATAAATCAGCATCC
AGTCTTTGTTGAGACCGCGTAGCCCGAAGGGTATGTTAGCTATCTAACAGCGTGGACTAACGGGCTGTAATAGCGCTCGGGTAAATATGGGTATATTTAGTCGTAGG

6,000 6,020 6,040 6,060 6,080

ATGTTGGAATTTAATCGCGGCCTCGAGCAAGACGTTTCCCGTTGAATATGGCTCATAACACCCCTTGTATTACTGTTTATGTAAGCAGACAGTTTTATTGTTCATGA
TACAACCTTAAATTAGCGCCGGAGCTCGTTCTGCAAAGGGCAACTTATACCGAGTATTGTGGGGAACATAATGACAAATACATTCGTCTGTCAAAATAACAAGTACT

6,100 6,120 6,140 6,160 6,180 6,200

TGATATATTTTTATCTTGTGCAATGTAACATCAGAGATTTTGAGACACAACGTGGCTTTCCCCCCCCCCCCTGCAGGTCGGCATCACCGGCGCCACAGGTGCGGTTG
ACTATATAAAAATAGAACACGTTACATTGTAGTCTCTAAAACTCTGTGTTGCACCGAAAGGGGGGGGGGGGACGTCCAGCCGTAGTGGCCGCGGTGTCCACGCCAAC

6,220 6,240 6,260 6,280 6,300

CTGGCGCCTATATCGCCGACATCACCGATGGGGAAGATCGGGCTCGCCACTTCGGGCTCATGAGCGCTTGTTTCGGCGTGGGTATGGTGGCAGGCCCCGTGGCCGGG
GACCGCGGATATAGCGGCTGTAGTGGCTACCCCTTCTAGCCCGAGCGGTGAAGCCCGAGTACTCGCGAACAAAGCCGCACCCATACCACCGTCCGGGGCACCGGCCC

6,320 6,340 6,360 6,380 6,400 6,420

GGACTGTTGGGCGCCATCTCCTTGCATGCACCATTCCTTGCGGCGGCGGTGCTCAACGGCCTCAACCTACTACTGGGCTGCTTCCTAATGCAGGAGTCGCATAAGGG
CCTGACAACCCGCGGTAGAGGAACGTACGTGGTAAGGAACGCCGCCGCCACGAGTTGCCGGAGTTGGATGATGACCCGACGAAGGATTACGTCCTCAGCGTATTCCC

6,440 6,460 6,480 6,500 6,520

AGAGCGTCGAGTATCTATGATTGGAAGTATGGGAATGGTGATACCCGCATTCTTCAGTGTCTTGAGGTCTCCTATCAGATTATGCCCAACTAAAGCAACCGGAGGAG
TCTCGCAGCTCATAGATACTAACCTTCATACCCTTACCACTATGGGCGTAAGAAGTCACAGAACTCCAGAGGATAGTCTAATACGGGTTGATTTCGTTGGCCTCCTC

6,540 6,560 6,580 6,600 6,620

GAGATTTCATGGTAAATTTCTCTGACTTTTGGTCATCAGTAGACTCGAACTGTGAGACTATCTCGGTTATGACAGCAGAAATGTCCTTCTTGGAGACAGTAAATGAA
CTCTAAAGTACCATTTAAAGAGACTGAAAACCAGTAGTCATCTGAGCTTGACACTCTGATAGAGCCAATACTGTCGTCTTTACAGGAAGAACCTCTGTCATTTACTT

6,640 6,660 6,680 6,700 6,720 6,740

GTCCCACCAATAAAGAAATCCTTGTTATCAGGAACAAACTTCTTGTTTCGAACTTTTTCGGTGCCTTGAACTATAAAATGTAGAGTGGATATGTCGGGTAGGAATGG
CAGGGTGGTTATTTCTTTAGGAACAATAGTCCTTGTTTGAAGAACAAAGCTTGAAAAAGCCACGGAACTTGATATTTTACATCTCACCTATACAGCCCATCCTTACC

6,760 6,780 6,800 6,820 6,840

AGCGGGCAAATGCTTACCTTCTGGACCTTCAAGAGGTATGTAGGGTTTGTAGATACTGATGCCAACTTCAGTGACAACGTTGCTATTTCGTTCAAACCATTCCGAAT
TCGCCCGTTTACGAATGGAAGACCTGGAAGTTCTCCATACATCCCAAACATCTATGACTACGGTTGAAGTCACTGTTGCAACGATAAAGCAAGTTTGGTAAGGCTTA

6,860 6,880 6,900 6,920 6,940

CCAGAGAAATCAAAGTTGTTTGTCTACTATTGATCCAAGCCAGTGCGGTCTTGAAACTGACAATAGTGTGCTCGTGTTTTGAGGTCATCTTTGTATGAATAAATCTA
GGTCTCTTTAGTTTCAACAAACAGATGATAACTAGGTTCGGTCACGCCAGAACTTTGACTGTTATCACACGAGCACAAAACTCCAGTAGAAACATACTTATTTAGAT

6,960 6,980 7,000 7,020 7,040 7,060

GTCTTTGATCTAAATAATCTTGACGAGCCAAGGCGATAAATACCCAAATCTAAAACTCTTTTAAAACGTTAAAAGGACAAGTATGTCTGCCTGTATTAAACCCCAAA
CAGAAACTAGATTTATTAGAACTGCTCGGTTCCGCTATTTATGGGTTTAGATTTTGAGAAAATTTTGCAATTTTCCTGTTCATACAGACGGACATAATTTGGGGTTT

7,080 7,100 7,120 7,140 7,160

TCAGCTCGTAGTCTGATCCTCATCAACTTGAGGGGCACTATCTTGTTTTAGAGAAATTTGCGGAGATGCGATATCGAGAAAAAGGTACGCTGATTTTAAACGTGAAA
AGTCGAGCATCAGACTAGGAGTAGTTGAACTCCCCGTGATAGAACAAAATCTCTTTAAACGCCTCTACGCTATAGCTCTTTTTCCATGCGACTAAAATTTGCACTTT

7,180 7,200 7,220 7,240 7,260

```
TTTATCTCAAGATCTCTGCCTCGCGCGTTTCGGTGATGACGGTGAAAACCTCTGACACATGCAGCTCCCGGAGACGGTCACAGCTTGTCTGTAAGCGGATGCCGGGA
AAATAGAGTTCTAGAGACGGAGCGCGCAAAGCCACTACTGCCACTTTTGGAGACTGTGTACGTCGAGGGCCTCTGCCAGTGTCGAACAGACATTCGCCTACGGCCCT
 7,280      7,300      7,320      7,340      7,360      7,380

GCAGACAAGCCCGTCAGGGCGCGTCAGCGGGTGTTGGCGGGTGTCGGGGCGCAGCCATGACCCAGTCACGTAGCGATAGCGGAGTGTATACTGGCTTAACTATGCGG
CGTCTGTTCGGGCAGTCCCGCGCAGTCGCCCACAACCGCCCACAGCCCCGCGTCGGTACTGGGTCAGTGCATCGCTATCGCCTCACATATGACCGAATTGATACGCC
 7,400      7,420      7,440      7,460      7,480

                              NdeI
CATCAGAGCAGATTGTACTGAGAGTGCACCATATGCGGTGTGAAATACCGCACAGATGCGTAAGGAGAAAATACCGCATCAGGCGCTCTTCCGCTTCCTCGCTCACT
GTAGTCTCGTCTAACATGACTCTCACGTGGTATACGCCACACTTTATGGCGTGTCTACGCATTCCTCTTTTATGGCGTAGTCCGCGAGAAGGCGAAGGAGCGAGTGA
 7,500      7,520      7,540      7,560      7,580

GACTCGCTGCGCTCGGTCGTTCGGCTGCGGCGAGCGGTATCAGCTCACTCAAAGGCGGTAATACGGTTATCCACAGAATCAGGGGATAACGCAGGAAAGAACATGTG
CTGAGCGACGCGAGCCAGCAAGCCGACGCCGCTCGCCATAGTCGAGTGAGTTTCCGCCATTATGCCAATAGGTGTCTTAGTCCCCTATTGCGTCCTTTCTTGTACAC
 7,600      7,620      7,640      7,660      7,680      7,700

AGCAAAAGGCCAGCAAAAGGCCAGGAACCGTAAAAAGGCCGCGTTGCTGGCGTTTTTCCATAGGCTCCGCCCCCCTGACGAGCATCACAAAAATCGACGCTCAAGTC
TCGTTTTCCGGTCGTTTTCCGGTCCTTGGCATTTTTCCGGCGCAACGACCGCAAAAAGGTATCCGAGGCGGGGGGACTGCTCGTAGTGTTTTTAGCTGCGAGTTCAG
                                          ColE1 origin
 7,720      7,740      7,760      7,780      7,800

AGAGGTGGCGAAACCCGACAGGACTATAAAGATACCAGGCGTTTCCCCCTGGAAGCTCCCTCGTGCGCTCTCCTGTTCCGACCCTGCCGCTTACCGGATACCTGTCC
TCTCCACCGCTTTGGGCTGTCCTGATATTTCTATGGTCCGCAAAGGGGGACCTTCGAGGGAGCACGCGAGAGGACAAGGCTGGGACGGCGAATGGCCTATGGACAGG
                                          ColE1 origin
 7,820      7,840      7,860      7,880      7,900

GCCTTTCTCCCTTCGGGAAGCGTGGCGCTTTCTCAATGCTCACGCTGTAGGTATCTCAGTTCGGTGTAGGTCGTTCGCTCCAAGCTGGGCTGTGTGCACGAACCCCC
CGGAAAGAGGGAAGCCCTTCGCACCGCGAAAGAGTTACGAGTGCGACATCCATAGAGTCAAGCCACATCCAGCAAGCGAGGTTCGACCCGACACACGTGCTTGGGGG
                                          ColE1 origin
 7,920      7,940      7,960      7,980      8,000      8,020

CGTTCAGCCCGACCGCTGCGCCTTATCCGGTAACTATCGTCTTGAGTCCAACCCGGTAAGACACGACTTATCGCCACTGGCAGCAGCCACTGGTAACAGGATTAGCA
GCAAGTCGGGCTGGCGACGCGGAATAGGCCATTGATAGCAGAACTCAGGTTGGGCCATTCTGTGCTGAATAGCGGTGACCGTCGTCGGTGACCATTGTCCTAATCGT
                                          ColE1 origin
 8,040      8,060      8,080      8,100      8,120

GAGCGAGGTATGTAGGCGGTGCTACAGAGTTCTTGAAGTGGTGGCCTAACTACGGCTACACTAGAAGGACAGTATTTGGTATCTGCGCTCTGCTGAAGCCAGTTACC
CTCGCTCCATACATCCGCCACGATGTCTCAAGAACTTCACCACCGGATTGATGCCGATGTGATCTTCCTGTCATAAACCATAGACGCGAGACGACTTCGGTCAATGG
                                          ColE1 origin
 8,140      8,160      8,180      8,200      8,220
```

TTCGGAAAAAGAGTTGGTAGCTCTTGATCCGGCAAACAAACCACCGCTGGTAGCGGTGGTTTTTTTGTTTGCAAGCAGCAGATTACGCGCAGAAAAAAAGGATCTCA
AAGCCTTTTTCTCAACCATCGAGAACTAGGCCGTTTGTTTGGTGGCGACCATCGCCACCAAAAAAACAAACGTTCGTCGTCTAATGCGCGTCTTTTTTTCCTAGAGT

ColE1 origin

8,240 8,260 8,280 8,300 8,320 8,340

AGAAGATCCTTTGATCTTTTCTACGGGGTCTGACGCTCAGTGGAACGAAAACTCACGTTAAGGGATTTTGGTCATGAGATTATCAAAAAGGATCTTCACCTAGATCC
TCTTCTAGGAAACTAGAAAAGATGCCCCAGACTGCGAGTCACCTTGCTTTTGAGTGCAATTCCCTAAAACCAGTACTCTAATAGTTTTTCCTAGAAGTGGATCTAGG

ColE1 origin

8,360 8,380 8,400 8,420 8,440

TTTTAAATTAAAAATGAAGTTTTAAATCAATCTAAAGTATATATGAGTAAACTTGGTCTGACAGTTACCAATGCTTAATCAGTGAGGCACCTATCTCAGCGATCTGT
AAAATTTAATTTTTACTTCAAAATTTAGTTAGATTTCATATATACTCATTTGAACCAGACTGTCAATGGTTACGAATTAGTCACTCCGTGGATAGAGTCGCTAGACA

AmpR

8,460 8,480 8,500 8,520 8,540 8,560

CTATTTCGTTCATCCATAGTTGCCTGACTCCCCGTCGTGTAGATAACTACGATACGGGAGGGCTTACCATCTGGCCCCAGTGCTGCAATGATACCGCGAGACCCACG
GATAAAGCAAGTAGGTATCAACGGACTGAGGGGCAGCACATCTATTGATGCTATGCCCTCCCGAATGGTAGACCGGGGTCACGACGTTACTATGGCGCTCTGGGTGC

AmpR

8,580 8,600 8,620 8,640 8,660

CTCACCGGCTCCAGATTTATCAGCAATAAACCAGCCAGCCGGAAGGGCCGAGCGCAGAAGTGGTCCTGCAACTTTATCCGCCTCCATCCAGTCTATTAATTGTTGCC
GAGTGGCCGAGGTCTAAATAGTCGTTATTTGGTCGGTCGGCCTTCCCGGCTCGCGTCTTCACCAGGACGTTGAAATAGGCGGAGGTAGGTCAGATAATTAACAACGG

AmpR

8,680 8,700 8,720 8,740 8,760

GGGAAGCTAGAGTAAGTAGTTCGCCAGTTAATAGTTTGCGCAACGTTGTTGCCATTGCTGCAGGCATCGTGGTGTCACGCTCGTCGTTTGGTATGGCTTCATTCAGC
CCCTTCGATCTCATTCATCAAGCGGTCAATTATCAAACGCGTTGCAACAACGGTAACGACGTCCGTAGCACCACAGTGCGAGCAGCAAACCATACCGAAGTAAGTCG

AmpR

8,780 8,800 8,820 8,840 8,860 8,880

TCCGGTTCCCAACGATCAAGGCGAGTTACATGATCCCCCATGTTGTGCAAAAAAGCGGTTAGCTCCTTCGGTCCTCCGATCGTTGTCAGAAGTAAGTTGGCCGCAGT
AGGCCAAGGGTTGCTAGTTCCGCTCAATGTACTAGGGGGTACAACACGTTTTTTCGCCAATCGAGGAAGCCAGGAGGCTAGCAACAGTCTTCATTCAACCGGCGTCA

AmpR

8,900 8,920 8,940 8,960 8,980

GTTATCACTCATGGTTATGGCAGCACTGCATAATTCTCTTACTGTCATGCCATCCGTAAGATGCTTTTCTGTGACTGGTGAGTACTCAACCAAGTCATTCTGAGAAT
CAATAGTGAGTACCAATACCGTCGTGACGTATTAAGAGAATGACAGTACGGTAGGCATTCTACGAAAAGACACTGACCACTCATGAGTTGGTTCAGTAAGACTCTTA

AmpR

9,000 9,020 9,040 9,060 9,080

AGTGTATGCGGCGACCGAGTTGCTCTTGCCCGGCGTCAACACGGGATAATACCGCGCCACATAGCAGAACTTTAAAAGTGCTCATCATTGGAAAACGTTCTTCGGGG
TCACATACGCCGCTGGCTCAACGAGAACGGGCCGCAGTTGTGCCCTATTATGGCGCGGTGTATCGTCTTGAAATTTTCACGAGTAGTAACCTTTTGCAAGAAGCCCC

AmpR

9,100 9,120 9,140 9,160 9,180 9,200

CGAAAACTCTCAAGGATCTTACCGCTGTTGAGATCCAGTTCGATGTAACCCACTCGTGCACCCAACTGATCTTCAGCATCTTTTACTTTCACCAGCGTTTCTGGGTG
GCTTTTGAGAGTTCCTAGAATGGCGACAACTCTAGGTCAAGCTACATTGGGTGAGCACGTGGGTTGACTAGAAGTCGTAGAAAATGAAAGTGGTCGCAAAGACCCAC

9,220 9,240 9,260 9,280 9,300

AGCAAAAACAGGAAGGCAAAATGCCGCAAAAAAGGGAATAAGGGCGACACGGAAATGTTGAATACTCATACTCTTCCTTTTTCAATATTATTGAAGCATTTATCAGG
TCGTTTTTGTCCTTCCGTTTTACGGCGTTTTTTCCCTTATTCCCGCTGTGCCTTTACAACTTATGAGTATGAGAAGGAAAAAGTTATAATAACTTCGTAAATAGTCC

9,320 9,340 9,360 9,380 9,400

GTTATTGTCTCATGAGCGGATACATATTTGAATGTATTTAGAAAAATAAACAAATAGGGGTTCCGCGCACATTTCCCCGAAAAGTGCCACCTGACGTCTAAGAAACC
CAATAACAGAGTACTCGCCTATGTATAAACTTACATAAATCTTTTTATTTGTTTATCCCCAAGGCGCGTGTAAAGGGGCTTTTCACGGTGGACTGCAGATTCTTTGG

9,420 9,440 9,460 9,480 9,500 9,520

ATTATTATCATGACATTAACCTATAAAAATAGGCGTATCACGAGGCCCTTTCGTCTTCAAGAATTAATTCTCATGTTTGACAGCTTATCATCGATAAGCTGACTCAT
TAATAATAGTACTGTAATTGGATATTTTTATCCGCATAGTGCTCCGGGAAAGCAGAAGTTCTTAATTAAGAGTACAAACTGTCGAATAGTAGCTATTCGACTGAGTA

9,540 9,560 9,580 9,600 9,620

GTTGGTATTGTGAAATAGACGCAGATCGGGAACACTGAAAAATAACAGTTATTATTCG
CAACCATAACACTTTATCTGCGTCTAGCCCTTGTGACTTTTTATTGTCAATAATAAGC

9,640 9,650 9,660 9,670 9,680
